# *Aloe vera* –Honey Edible Coating Prolongs Shelf Life and Preserves Quality of Refrigerated Fresh‐Cut Mango ( *Mangifera indica* )

**DOI:** 10.1002/fsn3.72447

**Published:** 2026-10-03

**Authors:** Tupura Khatun, Md. Asaduzzaman

**Affiliations:** ^1^ Department of Mathematics, Chittagong College National University Chittagong Bangladesh; ^2^ School of Food Science and Technology Jiangnan University Wuxi Jiangsu Province China; ^3^ State Key Laboratory of Food Science and Resources Jiangnan University Wuxi China; ^4^ Department of Food Engineering and Nutrition Science State University of Bangladesh Dhaka Bangladesh

**Keywords:** *Aloe vera*, mango, mixed flower honey, refrigeration

## Abstract

Postharvest deterioration of fresh‐cut mangoes limits shelf life, marketability, and nutritional quality, creating a need for safe, sustainable preservation strategies. This study investigated the effectiveness of edible coatings formulated from *Aloe vera* gel and mixed flower honey in preserving the quality and extending the refrigerated shelf life of fresh‐cut local mangoes ( *Mangifera indica* ) from Bangladesh. Fresh‐cut mango cubes were coated with six formulations comprising an uncoated control, 100% *Aloe vera* gel, 100% mixed flower honey, and different binary combinations of *Aloe vera* gel and honey, and stored at below 4°C for 20 days. We evaluated physicochemical attributes, antioxidant compounds, color, firmness, microbial quality, and spoilage at 5‐day intervals. Compared with the uncoated control, the coatings significantly reduced moisture loss, microbial proliferation, and fruit spoilage while preserving firmness, color, vitamin C, titratable acidity, total soluble solids, total phenolic content, and total flavonoid content. However, contrary to the hypothesis that honey would synergistically enhance *Aloe vera* 's preservative effects, 100% *Aloe vera* gel consistently outperformed all honey‐containing formulations. Among all treatments, the 100% *Aloe vera* coating exhibited the highest preservation efficiency, reducing weight loss by approximately 48% and spoilage by 50% after 20 days of storage while maintaining the lowest microbial counts and the greatest retention of antioxidant compounds and physicochemical quality. Principal component and correlation analyses further confirmed that reduced moisture loss was strongly associated with improved firmness and antioxidant retention. These findings demonstrate that *Aloe vera* ‐based edible coatings, particularly 100% *Aloe vera* gel, provide an effective, environmentally friendly, and biodegradable approach for extending the shelf life and maintaining the quality of fresh‐cut mangoes. This technology offers promising potential for commercial postharvest preservation of fresh‐cut mangoes and other climacteric fruits while reducing postharvest losses and reliance on synthetic preservatives.

## Graphical abstract

Fresh‐cut mango cubes were dip‐coated with *Aloe vera* gel, mixed flower honey, or their blends and stored under refrigeration for 20 days. Among all formulations, the 100% *Aloe vera* coating formed the most effective protective barrier. Compared with uncoated mango, it reduced moisture loss by approximately 48% and spoilage by 50%, while maintaining firmness, color, vitamin C, phenolic and flavonoid contents, and the lowest microbial counts. Honey‐containing blends provided protection but did not improve the overall preservation performance of pure *Aloe vera* . *Aloe vera* gel is therefore a sustainable, biodegradable alternative to synthetic preservatives for extending the refrigerated shelf life of fresh‐cut mango.

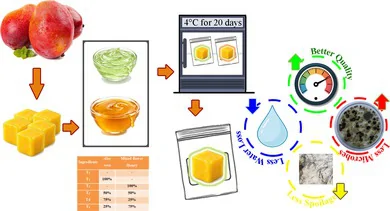

**Abbreviations**
DMRT: Duncan's Multiple Range Test
FN: firmness
HPMC: hydroxypropylmethylcellulose
LDPE: low‐density polyethylene
PAL: phenylalanine ammonia‐lyase
PCA: plate count agar
PDA: potato dextrose agar
QC: quality control
TA: titratable acidity
TFC: total flavonoid content
TPC: total polyphenol content
TSS: total soluble solids

## 1 Introduction

Mango ( *Mangifera indica* ), a monoecious plant and the most economically important tropical fruit, belongs to the family Anacardiaceae and is widely cultivated worldwide. Annual mango production in Bangladesh, cultivated on almost 139 thousand hectares, exceeded 1872 thousand metric tons in 2025 (BBS 2025). Mangoes belong to the category of climacteric fruits, which are an excellent source of macronutrients such as carbohydrates and fiber but also micronutrients such as Vitamin A, B_9_ (Folic acid), C, and K, and minerals such as Iron, Magnesium, Calcium, Potassium, and Zinc, and other various antioxidants including carotenoids, phenols, and flavonoids, and are a highly perishable product (Kuldeep et al. 2025; Marlinda et al. 2021; Masibo and He 2009; Molla et al. 2020; Ramos et al. 2026). Because fresh‐cut ripe mangoes have high water activity in the postharvest stage, they are vulnerable to microbial and physiological spoilage, which affects their use for home consumption and export (Zhao et al. 2022). This is why fresh‐cut mangoes urgently need innovative preservation and storage techniques that are effective, nutritious, and sustainable.

New studies highlight the promise of plant‐derived substances and food coatings for postharvest fruit protection. *Aloe vera* and honey have strong antioxidant, antimicrobial, antifungal, and moisture‐ and carbon dioxide (CO_2_)‐retention capabilities, recognized not only today but also in ancient times (Roy et al. 2023). As an important ingredient of *Aloe vera* , which includes water, vitamins, glucomannans, sterols, and amino acids, it has been shown to limit bacterial growth, control respiration, and minimize oxidation‐induced browning in several fruits, such as limes and grapes. These studies show that mixed fruit honey has significant bioactive effects (Roy et al. 2023; Saha et al. 2023). It is a natural antioxidant and can substitute for synthetic antioxidants (Tlak Gajger et al. 2025). Saha et al. (2023) and Roy et al. (2023) found that an edible coating made from *Aloe vera* gel with honey did not change the taste, smell, and texture of the fruit (Roy et al. 2023; Saha et al. 2023).

Nevertheless, despite these positive outcomes, knowledge remains limited on the potential of combining natural compounds, such as *Aloe vera* and honey, to preserve fresh‐cut mango. Most existing studies have focused on analyzing the benefits of individual treatments; therefore, the effects of combining them on microbial safety, physiological attributes, and sensory characteristics of stored fruits remain unknown. Identifying optimal approaches for applying these natural compounds and establishing effective dosages could also contribute significantly to the development of postharvest preservation technologies. This study provides the first systematic comparison of pure versus blended *Aloe vera* –honey edible coatings on fresh‐cut local mango ( *Mangifera indica* ). While *Aloe vera* and honey have been independently or jointly applied to other climacteric fruits, it remains unknown whether honey addition enhances or compromises *Aloe vera* 's barrier properties in mango.

Plant‐based bio‐coatings have proven beneficial in preserving fruits after harvesting (Oliveira et al. 2025). Honey has antimicrobial properties that inhibit bacterial and fungal growth, as well as enzymes involved in discoloration and decomposition (Iorizzo et al. 2025; Todorov et al. 2025). Nonetheless, these agents face an obstacle when used independently: they may alter taste or inhibit only a narrow range of microbes (Saha et al. 2023).

Bioactive substances integrated into biodegradable coatings have already shown promising results as sustainable preservatives (Diogo Gonçalves et al. 2025). This research could help the nation preserve its produce and reduce carbon dioxide emissions from food waste. In this particular case, *Aloe vera* and mixed flower honey were used to create a semi‐permeable barrier on the fresh‐cut surface of the fruit. To date, no study has evaluated the comparative efficacy of pure versus blended *Aloe vera* –honey coatings on fresh‐cut mango quality during refrigerated storage. This study addresses that gap by systematically comparing six coating formulations to test the hypothesis that honey addition enhances *Aloe vera* 's barrier properties while also considering the alternative possibility that honey's hygroscopic nature and high monosaccharide content may compromise the polysaccharide film matrix and reduce coating efficacy.

## 2 Materials and Methods

### 2.1 Raw Materials, Chemicals, and Other Ingredients

Fully matured mangoes (Local Varieties: Ashwina, fully ripe, golden yellow‐colored pulp) were obtained from the local market at Kanchan Market, Purbachal, Dhaka, Bangladesh, near the laboratory, selected for their maturity, color, and being free from defects during the mid‐April to mid‐June 2025 season. *Aloe vera* (Local varieties: Barbadensis Miller), mixed flower honey (Local Brand: Real), and low‐density polyethylene (LDPE) zipper bags were used as packaging materials and were also sourced from the same local market. All chemicals used were collected from the Food Analytical & Processing Laboratories, State University of Bangladesh, Dhaka, Bangladesh.

### 2.2 Preparation of Samples and Edible Coating Solutions

#### 2.2.1 Preparation of Mango

Initially, all utensils, such as containers, chopping boards, and knives, were cleaned using a 70% ethanol solution. Thereafter, mangoes were washed in stages using tap water and allowed to dry naturally. They were thereafter peeled manually and sliced into cubes (2 × 2 × 2 cm).

#### 2.2.2 Preparation of Coating Materials

*Aloe vera* leaves were purchased from the local market situated in front of the State University of Bangladesh. The leaves were washed thoroughly and trimmed with a knife to remove any jagged edges. The top portion of the leaf was removed longitudinally, and then the *Aloe vera* gel was separated with a spoon from it. This was done to obtain homogeneous gel pieces, which were then blended completely using a commercial blender (Jaipan CM/L‐7360065, Japan) at high speed (18,000 rpm) for 3 min in 200 g per batches at ambient temperature to obtain a homogeneous slurry before passing through an 850‐μm sieve. After removing the fiber, the extract was pasteurized at 63°C for 15 min. The *Aloe vera* was prepared at varying concentrations according to the previous study by Saha et al. (2023). First, pour 100 mL of *Aloe vera* juice into the beaker. To this, 1% glycerol (C_3_H_5_(OH)_3_), 1.5% Carboxymethyl Cellulose (CMC), 1% acetic acid (CH_3_COOH), and 2% methanol (CH_3_OH) were added to the *Aloe vera* gel and stirred well. The mixed gel solution was then heated at 70°C for 45 min and rapidly cooled to ambient temperature. As in the *Aloe vera* gel procedure, 100 mL of mixed flower honey was taken in a beaker, and 1% glycerol, 1.5% CMC, 1% acetic acid, and 2% methanol were added (Table 1). The formulation was prepared by adding 1% glycerol, 1.5% CMC, 1% acetic acid, and 2% methanol, then stirring continuously. The mixed solution was then heated to 70°C and cooled to ambient temperature.

**TABLE 1:** Composition of the samples.

Ingredients	*Aloe vera*	Mixed flower honey	Glycerol	CMC	Acetic acid	Methanol
T_0_	—	—	1%	1.5%	1%	2%
T_1_	100%	—				
T_2_	—	100%				
T_3_	50%	50%				
T4	75%	25%				
T_5_	25%	75%				

The base matrix (1% glycerol, 1.5% CMC, 1% acetic acid, and 2% methanol) was selected, following Saha et al. (2023), to ensure film‐forming capacity, plasticization, and antimicrobial stability. Glycerol (1%, w/v) acted as a plasticizer to improve coating flexibility and prevent cracking; CMC (1.5%, w/v) served as a thickening agent and film former to enhance adhesion to the hydrophobic mango cuticle; acetic acid (1%, v/v) was included to lower pH and inhibit polyphenol oxidase activity; and methanol (2%, v/v) was used to improve solubilization of honey bioactives and ensure homogeneous dispersion. We selected five *Aloe vera* : honey ratios (100:0, 75:25, 50:50, 25:75, and 0:100) to create a gradient and determine whether honey synergistically enhances or dilutes *Aloe vera* 's barrier properties, as honey's hygroscopic nature might compromise the polysaccharide matrix at higher concentrations.

#### 2.2.3 Mango Coating Process

We collected the coating solution in a beaker. Then, we immersed cube‐cut mangoes in an *Aloe vera* and mixed flower honey coating solution with different concentrations of individual constituents for 2 min, followed by drying for 20 min under ambient conditions. The samples were separated into six groups according to the treatments, where 350 fruits were used in the experiments [treatments (6) × Replications (3) × fruits per replication (3) × sampling interval (6)]. Fruits were selected from a single lot and assigned random numbers. An independent researcher, not involved in subsequent analyses, allocated treatments to fruits to ensure blinding. Finally, coated mangoes were packaged in LDPE bags and stored in a completely randomized design (CRD) at > 4°C with 85% relative humidity for 20 days (Walton, WNT‐3D3‐GDEL‐DD, Bangladesh) (Figure 1). Each treatment consisted of three independent biological replicates. One biological replicate comprised an independently prepared batch of three fresh‐cut mangoes that underwent coating, packaging, storage, and subsequent analyses separately from the other batches. We obtained measurements from each biological replicate and used the mean values for statistical analysis, avoiding pseudo‐replication. We carried out quality evaluation and other relevant analyses every 5 days during a one‐month storage period. In the present study, 30 randomly selected fruits were used for both destructive and non‐destructive assays such as spoilage percentage and moisture loss percentage, and destructive tests, including pH, total titratable acidity, Total Soluble Solids (TSS), vitamin C, phenols, flavonoids, sensory evaluation, and total microbial count, mold, and yeast count. For destructive analyses, we used a fresh set of fruits each sampling day and performed all measurements in triplicate.

**FIGURE 1:**
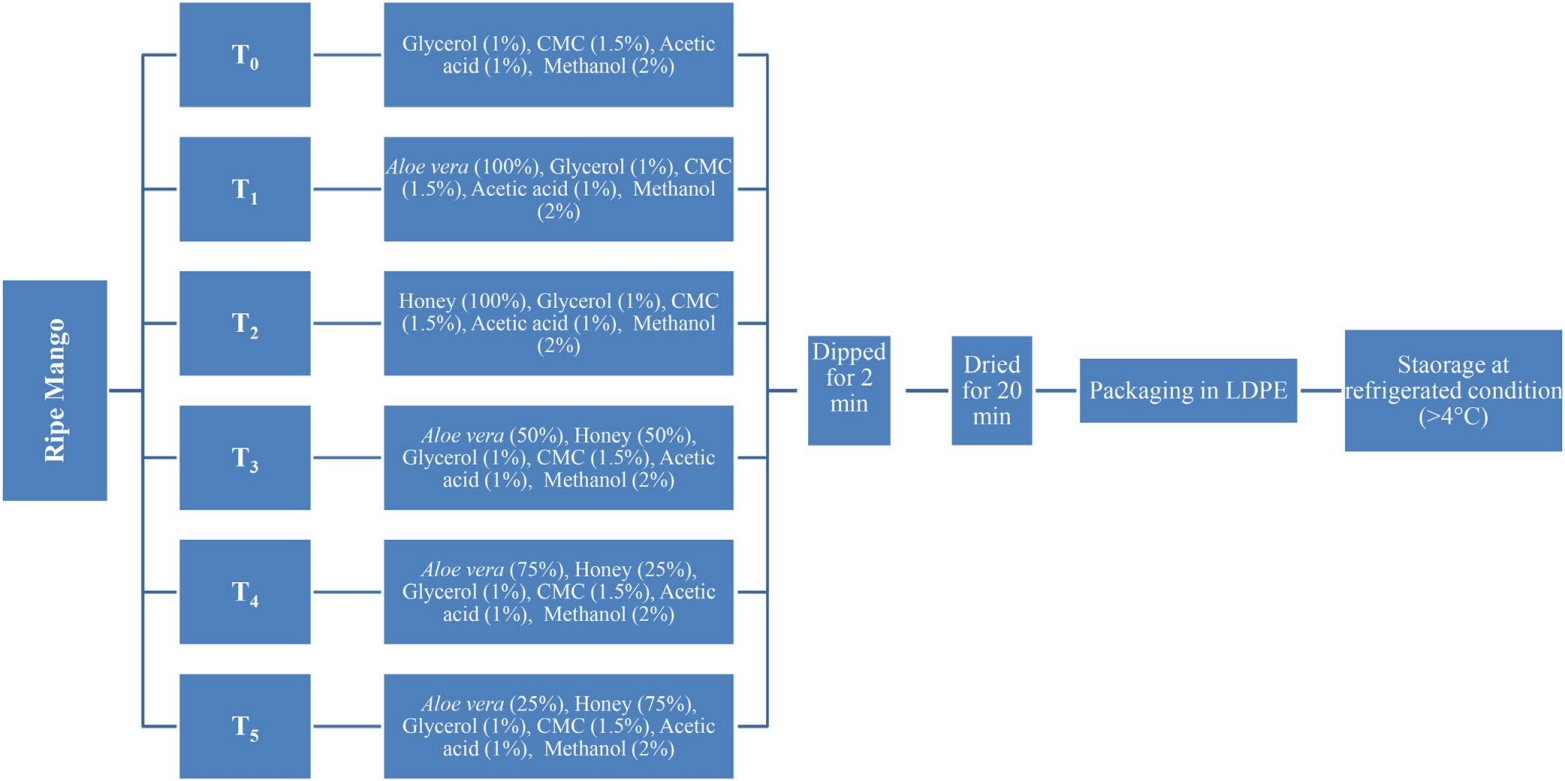
Flow diagram of the preparation of samples.

### 2.3 Proximate Composition of Fresh Mango

Moisture content of mango, *Aloe vera* , mixed flower honey, and coated mango was examined by the oven‐drying technique. Protein, fat, and ash content of raw ingredients and coated treatments were estimated using the Kjeldahl method, Soxhlet extraction, and muffle furnace method. The protein conversion factor was 6.25. The analytical methods for determining moisture (AOAC 934.06), ash (AOAC 940.26), protein (AOAC 920.152), fat (AOAC 996.06), and carbohydrates (AOAC 986.25) (Roy et al. 2023; Saha et al. 2023). We used Equation (1) to calculate carbohydrates.

(1) $$\text{Carbohydrates}=\text{Total weight}-\left(\text{Moisture}+\mathrm{Ash}+\text{Protein}+\mathrm{Fat}\right)$$

### 2.4 Fruit Physicochemical Analysis

#### 2.4.1 Moisture Loss

Moisture loss was expressed as percent leakage, calculated from weight loss during storage. We measured initial weight (Day 0) and weight throughout the storage period using a digital balance. The formula for percent moisture loss is presented below, where *W*_1_ and *W*_2_ are the initial and final weights, respectively.

(2) $$\text{Moisture loss}\;\left(\%\right)=\frac{W_{1}-W_{2}}{W_{1}}\times 100$$

#### 2.4.2 Firmness

Firmness was examined using a penetrometer (FDK 40, Wagner, USA) (Saha et al. 2023). Cut mango pieces were placed in the center of the platform, and the force required to penetrate the sample with the blade was measured. A puncture test was performed using a 3.5 mm stainless‐steel probe. The texture of the fruit was evaluated based on the maximum force (kilo‐Newton) required for probe penetration at three different locations of the samples. The resulting value was used to calculate mango firmness.

#### 2.4.3 Color

Changes in the color of cut mangoes were measured using a Colorimeter Minolta CM 2500d (Konica Minolta Optics Inc., Japan) (Akter et al. 2022; Khatun and Asaduzzaman 2025; Saha et al. 2023). We calibrated the instrument using standard white and black plates. Color coordinates (L*, a*, and b*) were calculated as the mean of three replications. The equation used for calculation is as follows,

(3) HueangleH=tan−1ba

(4) $$\text{Color differences}\;\left(∆E\right)={\left[{\left(∆L*\right)}^{2}+{\left(∆a*\right)}^{2}+{\left(∆b*\right)}^{2}\right]}^{1/2}$$

#### 2.4.4 Total Soluble Solids

Total soluble solids (TSS) were measured by AOAC Method 932.14 (AOAC 1998) with a digital refractometer (Saha et al. 2023).

#### 2.4.5 pH and Titratable Acidity

The pH values of the mangoes were measured using AOAC Method 981.12 (AOAC 1998) with a digital pH meter (Mettler Toledo, Switzerland) (Saha et al. 2023).

To determine titratable acidity (TA), 10 mL of fruit juice solution was diluted with 10 mL of distilled water and titrated with sodium hydroxide (0.1 N) to determine the color change of phenolphthalein (1%). The quantity of sodium hydroxide (NaOH) consumed was noted, and Equation (5) was applied to calculate the TA, where *V* is the volume (mL) of NaOH consumed, *N* is the normality of the NaOH (mol/L), *E* is the equivalent weight of the predominant acid (citric acid = 64.04 g/eq), and *W* = weight of the sample in the titrated aliquot (g).

(5) $$\text{Titratable acidity}=\frac{V\times N\times E\times 100}{W\times 1000}$$

#### 2.4.6 Flavor Index

The ratio was considered a flavor index (fruit maturity index) and determined by Equation (6).

(6) $$\text{Flavor index}=\frac{\text{Total soluble solids}}{\text{Titratable Acidity}}$$

#### 2.4.7 Vitamin C

The vitamin C in mangoes was determined according to AOAC 985.33 (titrimetric determination with 2,6‐dichloroindophenol; AOAC 1998) (Saha et al. 2023). Initially, 20 g of mangoes were mixed with 50 mL of a mixture of meta‐phosphoric acid and acetic acid. This mixture was then filtered using qualitative filter paper. Thereafter, 10 mL of the filtrate was placed in a conical flask, where the sample solution was titrated against a known concentration of 2,6‐dichloroindophenol. The volume of 2,6‐dichloroindophenol (0.025%) needed for the titration was recorded to calculate the vitamin C content in the sample, expressed as mg of ascorbic acid per gram of sample (wet basis). Vitamin C was calculated utilizing the equations:

(7) $$\mathrm{Dye}\;\text{factor}=\frac{0.5}{\text{Standard titration value}}$$

(8) VitaminCmg100gm=Titration value×dyefactor×100Weight of sample

#### 2.4.8 Total Polyphenol Content (TPC)

TPC was estimated using the protocol from the previous study (Akter et al. 2022; Asaduzzaman et al. 2013, 2025). Briefly, 1 g of the sample was extracted with 20 mL of 25% ethanol for 15 min, followed by filtration through Whatman No. 2 filter paper. The reaction mixture consisted of 1 mL of filtrate, 1 mL of Folin–Ciocalteu reagent, and 5 mL of Na_2_CO_3_. The mixture was incubated for 1 h at 25°C. We also prepared quality control (QC) samples for the accuracy test at five concentrations (10, 20, 30, 50, and 60 ppm) from serial dilutions of the fresh standard gallic acid stock solution, which should be used within 24 h of preparation. We used spectrophotometry (T80 UV/VIS, PG Instruments, United Kingdom) to measure phenolic compounds at 765 nm against a gallic acid calibration curve, with values presented as milligrams per gram of sample (mg GAE/g). Equation (9) is used to calculate the content of TPC, in which *Y* is the sample, while *X* is the TPC content (mg GAE/g).

(9) Y=0.0019×X+0.2066

$$R^{2}=0.995$$

#### 2.4.9 Total Flavonoid Content (TFC)

TFC was determined using a colorimetric method, with the Rutin standard curve applied according to the procedure outlined by Asaduzzaman et al. (2013) (Asaduzzaman et al. 2025; Khatun and Asaduzzaman 2025). The flavonoid extraction solution was prepared by dissolving the samples in methanol: 10 mg of each sample was dissolved in 10 mL of methanol, and 1 mL was pipetted into the test tube. Add 5 mL of distilled water and 0.1 mL of potassium acetate (CH3COOK) to the test tube, then leave the mixture to stand for 5 min. Then 0.3 mL of aluminum chloride (AlCl_3_) was added to the mixture and kept at 25°C for 30 min. QC samples were also prepared at six concentrations (0, 10, 20, 30, 40, and 50 μg/mL) to assess assay accuracy within 24 h. The absorbance of the sample flavonoids was analyzed using a spectrophotometer at 415 nm, and the TFC value was calculated from Equation (10), where *Y* represents the absorbance and *X* is the TFC content (mg/100 g).

(10) Y=0.0059×X+0.0527

$$R^{2}=0.981$$

### 2.5 Microbial Analysis

Total Viable Count (TVC) and total mold and yeast counts were determined by the standard pour plate method. Ten grams of minced treated mango was aseptically transferred into 90 mL of sterile physiological saline (0.85% NaCl) and homogenized to obtain a 10^−1^ dilution. For total aerobic bacteria, serial dilutions were prepared up to 10^−6^, and 0.1 mL of appropriate dilutions (10^−1^ to 10^−6^) was plated in triplicate on Plate Count Agar (PCA). Incubate plates aerobically at 37°C for 24–48 h. The dilution yielding 30–300 colonies was selected for enumeration. For molds and yeasts, plate 1.0 mL of the 10^−1^ dilution in duplicate on Potato Dextrose Agar (PDA) and incubate aerobically at 25°C for 3–5 days; enumerate plates yielding 10–100 colonies. The detection limit (DL) was calculated as 1 CFU divided by the product of the plated volume and the lowest dilution factor: 1.0 × 10^2^ CFU/g for total aerobic bacteria (0.1 mL of 10^−1^) and 1.0 × 10^1^ CFU/g for molds and yeasts (1.0 mL of 10^−1^). Counts below the DL were recorded as < DL. All microbial counts were log_10_‐transformed prior to statistical analysis (Mostofa et al. 2026; Saha et al. 2023).

### 2.6 Organoleptic Properties (Fruit Spoilage)

Two independent observers, blinded to treatment codes, visually assessed the degree of fruit spoilage. Spoilage signs, including fungal growth, surface discoloration, and textural breakdown (softening, translucency, or surface lesions), were recorded at each sampling interval. The spoilage percentage was calculated as the ratio of spoiled fruit pieces to the total number of fruit pieces analyzed per treatment, expressed as a percentage. Evaluations were conducted on Days 0, 5, 10, 15, 20, and 25 under consistent ambient lighting. No trained sensory panel or hedonic scaling was used; therefore, this study did not obtain formal taste, aroma, or texture‐liking scores (Roy et al. 2023).

### 2.7 Statistical Analysis

We analyzed the data using analysis of variance. We assessed the effects of coating treatments, storage intervals, and their interactions using the least significant difference test at a 5% significance level in R software (version 4.3.3). Data are presented as the mean ± standard deviation (SD) of three independent biological replicates.

## 3 Results and Discussion

### 3.1 Proximate Analyses

Table 2 indicates the Proximate analysis of the raw ingredients. As per observations made in the present experiment, the moisture, protein, fat, carbohydrates, ash, and TSS content in fresh mango were 88.28%, 1.05%, 1.15%, 7.09%, 2.43%, and 18.46 °Brix, respectively, which almost matches the findings of Nguyen et al. (2025), who reported, the moisture, protein, ash, fiber, and fat content in ripe fresh mango were 87.74%, 3.23%, 2.46%, 1.40%, and 2.60%, respectively; however, in *Aloe vera* the protein, moisture, carbohydrate, and ash contents were 0.13%, 98.5%, 1.25%, and 0.12%, respectively, which corresponds to the findings of Saha et al. (2023) for the same parameters, while the moisture, protein, ash, and carbohydrate contents in honey were 25.18%, 0.54%, 0.34%, and 73.76%, respectively (Saha et al. 2023). The differences may be due to fertilizer use, environmental variations, and the timing and place of cultivation (Mode et al. 2026).

**TABLE 2:** Proximate composition of mango, *Aloe vera,* and mixed flower honey.

Sample	Proximate composition (%)					
	Moisture	Protein	Fat	Carbohydrate	Ash	TSS (°Brix)
Mango	88.28 ± 0.39	1.05 ± 0.03	1.15 ± 0.04	7.09 ± 0.34	2.43 ± 0.08	18.46 ± 0.08
*Aloe vera*	98.5 ± 0.08	0.13 ± 0.01	—	1.25 ± 0.09	0.12 ± 0.02	1.15 ± 0.01
Mixed flower honey	25.18 ± 0.25	0.54 ± 0.01	0.18 ± 0.01	73.76 ± 0.24	0.34 ± 0.01	70 ± 0.28

*Note:* Mean ± standard deviation.

### 3.2 Physico‐Chemical Properties

#### 3.2.1 Moisture Loss

Figure 2 shows a significant (*p* ≤ 0.05) increase in moisture loss across all treatments throughout the storage period. Two‐way ANOVA revealed that treatment (*F*_5,60_ = 187.1, *p* < 0.0001), storage time (*F*_4,60_ = 1420, *p* < 0.0001), and their interaction (*F*_20,60_ = 28.86, *p* < 0.0001) significantly influenced moisture loss. Post hoc analysis (Tukey's HSD) indicated that at Day 20 (Table S1), T_1_ (*fresh‐cut mangoes coated with 100% Aloe vera*; 14.28% ± 0.42%) exhibited significantly lower moisture loss than all other treatments (*p* < 0.05), while T_0_ (control; 27.80% ± 0.69%) was significantly higher than the other samples (Table S1), *indicating suppressed respiration and transpiration*. This effect is consistent with the formation of a semi‐permeable film that limits water vapor and gas exchange, thereby lowering metabolic activity and delaying senescence. Similar moisture‐retention benefits of *Aloe vera* –based coatings have been reported for pineapple, guava, and ber fruits, supporting the generalizability of this mechanism across climacteric and non‐climacteric produce. The additional reduction in moisture loss under refrigeration likely reflects synergistic effects of low temperature and coating barrier properties, which together decrease water vapor pressure gradients and microbial‐driven degradation (Nasrin et al. 2026; Roy et al. 2023; Saha et al. 2023). The decrease in the percentage of moisture loss could also be attributed to a low temperature. As moisture is important for fruit quality, any loss will greatly contribute to spoilage. In some cases, however, controlled drying can improve the taste and quality of fruits. The process is mainly achieved through a semi‐permeable coating, which closes surface cracks to prevent water loss (Caner et al. 2026; Guo et al. 2026).

**FIGURE 2:**
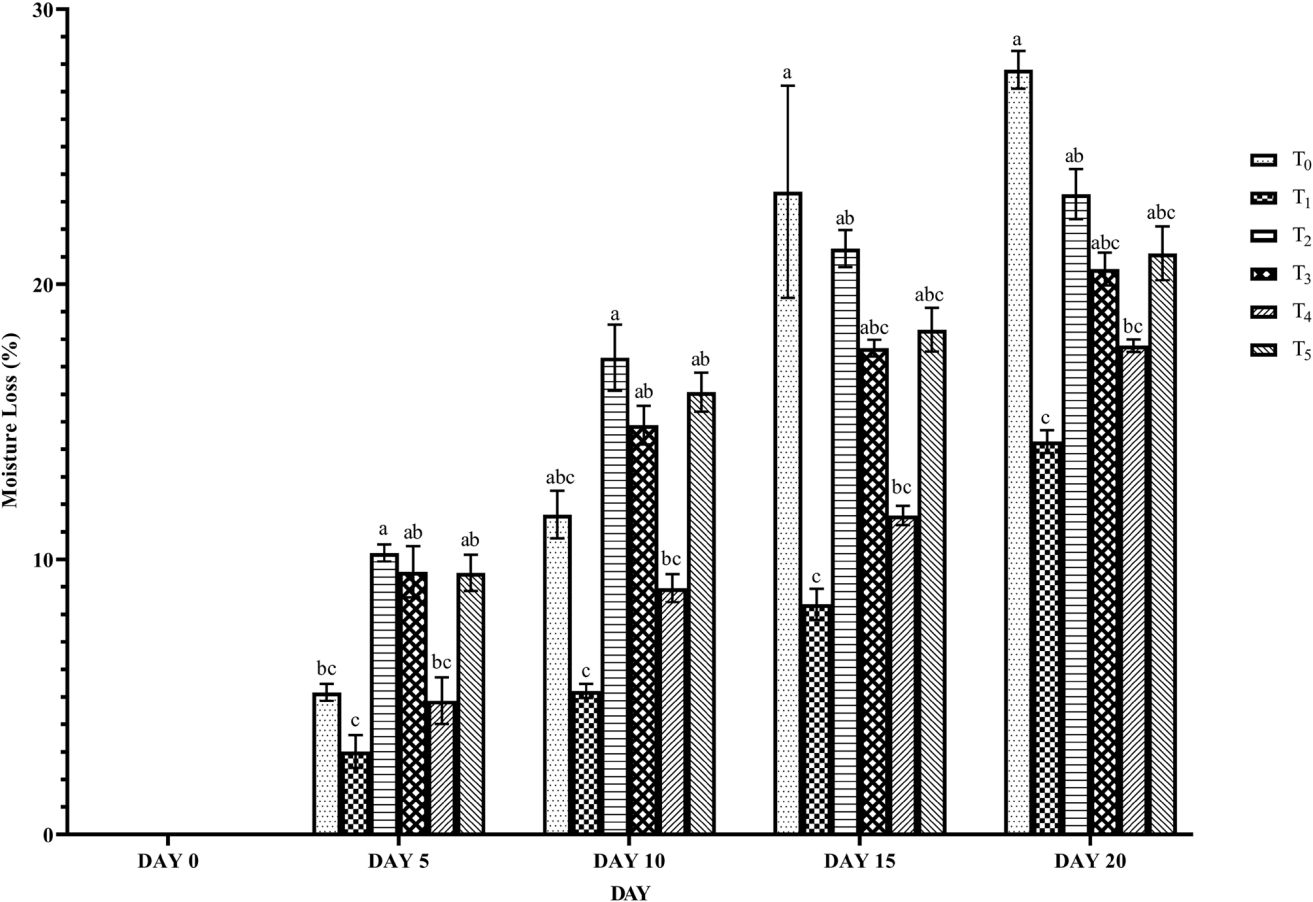
Effect of *Aloe vera* and mixed flower honey coating on juice leakage during refrigerated storage. Values are mean ± SD (*n* = 3). Different superscript letters indicate significant differences among treatments within the same storage time (*p* < 0.05) according to DMRT. Two‐way ANOVA showed significant effects of treatment (T), time (D), and their interaction (T × D) at *p* < 0.05, unless otherwise noted. T_0_ is our control untreated mango, T_1_ is mango coated with 100% *Aloe vera*, T_2_ is mango coated with 100% mixed flower honey, T_3_ is mango coated with 50% mixed flower honey and 50% *Aloe vera*, T_4_ is mango coated with 25% mixed flower honey and 75% *Aloe vera*, and T_5_ is mango coated with 75% mixed flower honey and 25% *Aloe vera* .

Fruits cubes without coatings showed the highest levels of decay after 20 days due to increased microbial activity. These are known to increase the rate of degradation and reduce the quality of the produce. *Aloe vera* and mixed flower honey coatings have antiseptic properties because their chemicals affect the cell walls of microbes, preventing them from functioning normally. Films containing guar gum, alone or blended with essential oils, show high levels of effectiveness against various microbes. In addition, higher levels of antioxidant content in coatings not only ensure protection against physiological stress but also improve the fruits' resistance against microbes.

Notably, 100% *Aloe vera* consistently outperformed all honey‐containing formulations, including the T_3_ (50:50) and T_4_ (75:25) blends. This suggests that while honey possesses independent antimicrobial and antioxidant properties, its hygroscopic nature and high reducing sugar content may increase surface water activity when combined with *Aloe vera* gel, compromising the semi‐permeable barrier that suppresses respiration and transpiration. Consequently, the addition of honey to *Aloe vera* gel appears to dilute the film‐forming capacity of the polysaccharide‐rich *Aloe vera* matrix rather than synergistically enhancing it. This finding highlights that coating efficacy is fruit‐matrix specific and that the assumption that ‘more bioactive ingredients yield better preservation’ does not hold for fresh‐cut mango under refrigerated conditions.

#### 3.2.2 Firmness

Firmness is an essential characteristic of consumer acceptability in fresh fruits and depends on water content and metabolism. Fruit cubes become soft during extended storage. In this study, we assessed the firmness of uncoated and coated fresh‐cut mangoes and found that coated fresh‐cut mangoes were firmer than uncoated fresh‐cut mangoes (Table 3). In this experiment, two‐way ANOVA revealed that treatment (*F*_5,60_ = 23.92, *p* < 0.0001), storage time (*F*_4,60_= 45.31, *p* < 0.0001), and their interaction (*F*_20,60_ = 2.88, *p* = 0.0008) significantly influenced firmness (Table S2). Post hoc analysis (Tukey's HSD) indicated that the 100% Aloe vera coating and the T_4_ (75% *Aloe vera* + 25% mixed flower honey) mixture provided better firmness than other coating formulas, at greater than 90% and 71%, respectively. For T2, T4, and T5, firmness was poor due to high moisture loss (41%, 35%, and 29%, respectively). Similarly, Jama et al. (2026) mentioned that gellan‐ and alginate‐based edible coatings had a substantial effect on preserving the firmness of fresh‐cut apples and melons (Jama et al. 2026). Moreover, Seyedi et al. (2026) noted that *Aloe vera* gel could reduce weight loss, which also affects firmness (Seyedi et al. 2026).

**TABLE 3:** Effect of *Aloe vera* and mixed flower honey coating on firmness during refrigerated storage.

Treatment (*N*)	Storage duration (Day)				
	D_0_	D_5_	D_10_	D_15_	D_20_
T_0_	^A^6.20 ± 0.31^a^	^B^4.79 ± 0.41^b^	^BC^3.78 ± 0.65^b^	^D^2.59 ± 0.33^b^	^CD^2.78 ± 0.40^b^
T_1_	^A^6.07 ± 0.25^a^	^A^5.98 ± 0.37^a^	^A^5.95 ± 0.29^a^	^AB^5.63 ± 0.34^a^	^B^5.30 ± 0.58^a^
T_2_	^A^5.91 ± 0.30^a^	^B^4.97 ± 0.18^b^	^BC^4.81 ± 0.99^ab^	^BC^4.42 ± 0.25^a^	^C^3.92 ± 0.34^ab^
T_3_	^A^5.83 ± 0.90^a^	^AB^5.24 ± 0.31^ab^	^BC^4.56 ± 0.78^ab^	^AB^4.85 ± 0.50^a^	^C^3.59 ± 0.26^ab^
T_4_	^A^6.37 ± 0.26^a^	^B^5.37 ± 0.42^ab^	^B^4.91 ± 0.31^ab^	^B^5.21 ± 0.30^a^	^B^4.76 ± 0.20^a^
T_5_	^A^5.74 ± 0.46^a^	^B^4.70 ± 0.35^b^	^B^4.74 ± 1.03^ab^	^B^4.20 ± 0.23^ab^	^B^3.77 ± 0.33^ab^

*Note:* a–c means followed by different superscript alphabets in each column are significantly different (*p* < 0.05) among different storage durations within the same temperature. A–D means followed by different superscript alphabets in each row are significantly different (*p* < 0.05) among different treatments. Values are mean ± SD (*n* = 3). Different superscript letters indicate significant differences among treatments within the same storage time (*p* < 0.05) according to DMRT. Two‐way ANOVA showed significant effects of treatment (T), time (D), and their interaction (T × D) at *p* < 0.05, unless otherwise noted. T_0_ is our control untreated mango, T_1_ is mango coated with 100% *Aloe vera*, T_2_ is mango coated with 100% mixed flower honey, T_3_ is mango coated with 50% mixed flower honey and 50% *Aloe vera*, T_4_ is mango coated with 25% mixed flower honey and 75% *Aloe vera*, and T_5_ is mango coated with 75% mixed flower honey and 25% *Aloe vera* .

These differences indicate that *Aloe vera* gel formed a more effective semi‐permeable film than honey‐containing blends. Such coatings restrict oxygen transfer and slow respiration, thereby delaying the enzymatic breakdown of pectin and hemicellulose that causes softening in fresh‐cut fruit (Yang et al. 2026). Similar firmness retention has been reported for *Aloe vera* coatings on pineapple, guava, ber, and kiwifruit, and for other polysaccharide coatings on fresh‐cut apple, melon, and mango (Jama et al. 2026; Parente et al. 2026; Saha et al. 2023; Seyedi et al. 2026). Honey did not improve this effect. Its hygroscopic sugars likely weakened the polysaccharide matrix, so T_2_, T_3_, and T_5_ softened faster than T_1_. Firmness was therefore governed mainly by coating composition and storage time, not by moisture loss alone. The shelf‐life of fresh‐cut mangoes is critical for consumer willingness and supply chain sustainability. Untreated fresh‐cut mangoes are highly perishable, undergoing rapid physiological degradation and microbial spoilage due to surface exposure and loss of protective cuticle layers. Therefore, applying edible coatings is considered a primary strategy to slow these spoilage mechanisms.

#### 3.2.3 Total Soluble Solids (TSS) and Titratable Acidity (TA)

As storage duration increased, TSS concentration also increased (Figure 3A), rising to 1.23%, 1.65%, 3.83%, and 6.78% on Days 5, 10, 15, and 20, respectively, compared with Day 0. For TSS, the two‐way ANOVA revealed that treatment (*F*_5,60_ = 210.2, *p* < 0.0001), storage time (*F*_4,60_ = 113.9, *p* < 0.0001), and their interaction (*F*_20,60_ = 5.751, *p* < 0.0001) significantly influenced TSS (Table S3). The effect of treated samples was more pronounced than in untreated samples at Days 15 and 20 in terms of TSS concentration. Neither the concentration of the *Aloe vera* gel nor the mixed flower honey had a significant effect on the TA. The significant effect of storage time was attributed to the increment in TA. TA dropped by 20%, 27%, 68.9%, and 65% at Days 5, 10, 15, and 20, respectively (Figure 3B).

**FIGURE 3A:**
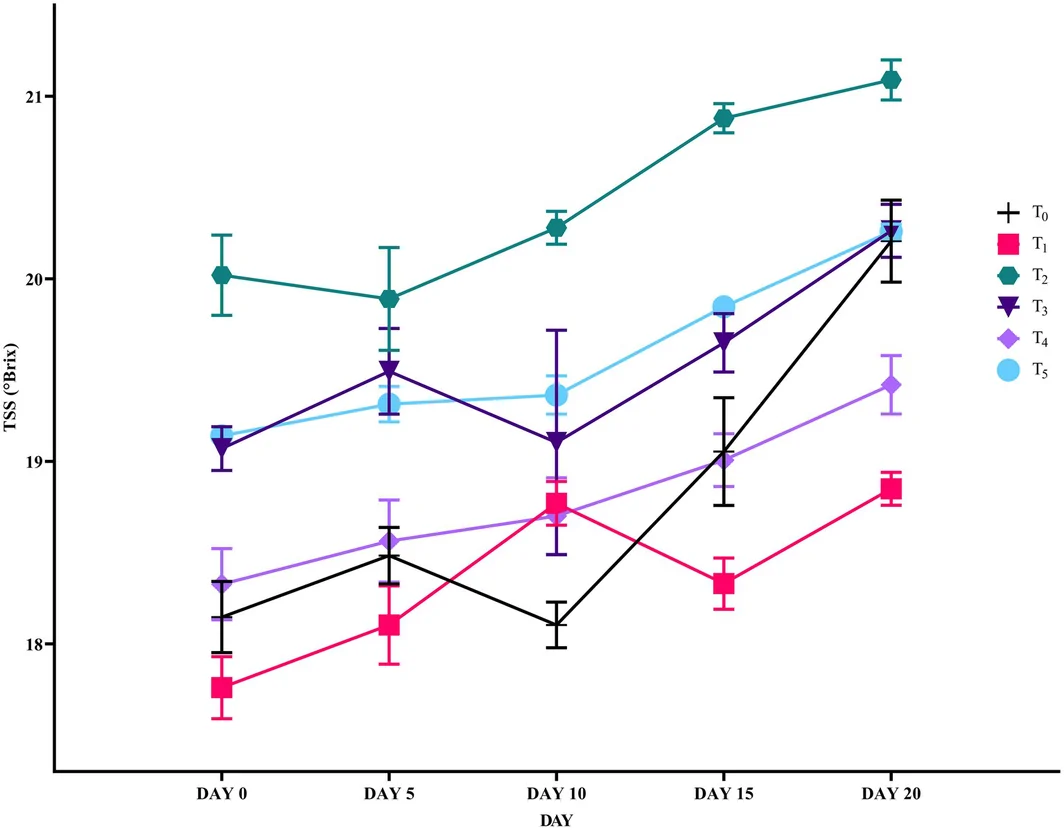
Effect of *Aloe vera* and mixed flower honey coating on TSS during refrigerated storage. Values are mean ± SD (*n* = 3). Two‐way ANOVA showed significant effects of treatment (T), time (D), and their interaction (T × D) at *p* < 0.05, unless otherwise noted. T_0_ is our control untreated mango, T_1_ is mango coated with 100% *Aloe vera*, T_2_ is mango coated with 100% mixed flower honey, T_3_ is mango coated with 50% mixed flower honey and 50% *Aloe vera*, T_4_ is mango coated with 25% mixed flower honey and 75% *Aloe vera*, and T_5_ is mango coated with 75% mixed flower honey and 25% *Aloe vera* .

**FIGURE 3B:**
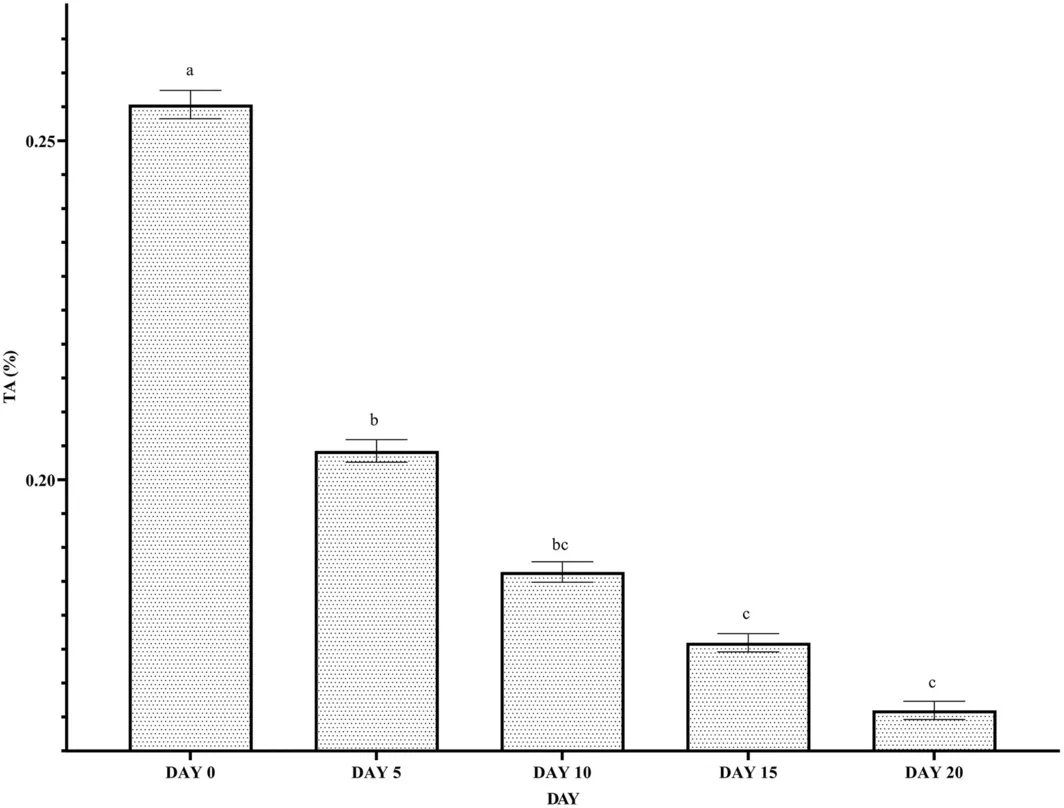
Effect of *Aloe vera* and mixed flower honey coating on TA during refrigerated storage. Values are presented as mean ± standard deviation (SD). Bars with different lowercase letters (a–c) are significantly different according to Duncan’s multiple range test at *p* < 0.05.

Storage time clearly increased the flavor index, as measured by the TSS/TA ratio. The TSS/TA ratio increased in similar folds on Days 0, 5, 10, 15, and 20 compared to Day 0, despite differences in the increments for each period. For TSS/TA ratio, the two‐way ANOVA revealed that treatment (*F*_5,60_ = 153.9, *p* < 0.0001), storage time (*F*_4,60_ = 4056, *p* < 0.0001), and their interaction (*F*_20,60_ = 5.463, *p* < 0.0001) significantly influenced TSS/TA (Table S4). The ratio in treated samples was always higher than in untreated samples throughout the storage period, despite changes in both. The higher ratios of treated samples indicate a better balance between sweetness and acidity in favor of taste. Post hoc comparison showed that the uncoated control (T0T_0T0) differed significantly from the coated treatments for TSS at Days 15 and 20 (*p* < 0.05p < 0.05p < 0.05), whereas the coating formulations did not differ significantly from one another for TA. For the TSS/TA ratio, T_3_ (Mango coated with 50% mixed flower honey and 50% *Aloe vera* ) increased the TSS/TA ratio by 16% on Day 20 (Figure 3C).

**FIGURE 3C:**
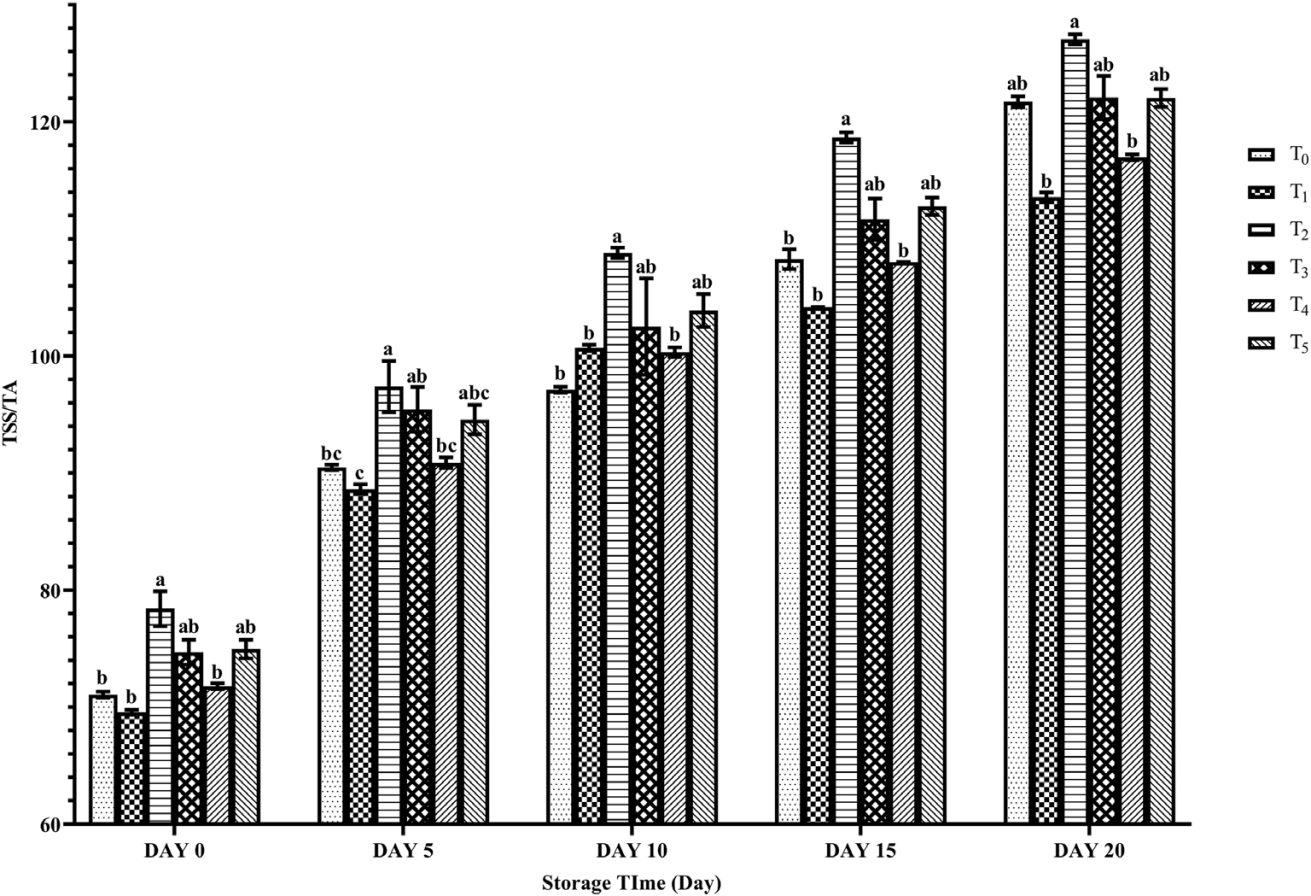
Effect of *Aloe vera* and mixed flower honey coating on TSS/TA ratio during refrigerated storage. Values are mean ± SD (*n* = 3). Different superscript letters indicate significant differences among treatments within the same storage time (*p* < 0.05) according to DMRT. Two‐way ANOVA showed significant effects of treatment (T), time (D), and their interaction (T × D) at *p* < 0.05, unless otherwise noted. T_0_ is our control untreated mango, T_1_ is mango coated with 100% *Aloe vera*, T_2_ is mango coated with 100% mixed flower honey, T_3_ is mango coated with 50% mixed flower honey and 50% *Aloe vera*, T_4_ is mango coated with 25% mixed flower honey and 75% *Aloe vera*, and T_5_ is mango coated with 75% mixed flower honey and 25% *Aloe vera* .

The conversion of starch to sugars during unripe‐to‐ripe maturation increases TSS, a process accelerated by increased ethylene production (Li, Wang, et al. 2026). Fruit cubes coated with edible films show slower respiration rates and altered internal atmospheres characterized by reduced oxygen and ethylene and increased carbon dioxide, which helps limit the rise in TSS compared to uncoated fruits, especially toward the end of storage time (Roy et al. 2023; Saha et al. 2023). Chemical changes within plant tissues greatly affect fruit quality as they progress through their developmental cycle. The soluble solids‐to‐acidity ratio is key to deciding when to harvest and how to use the fruit, especially for fruits that continue to develop after harvest. Flavor depends on sugars, organic acids, phenolics, and aromatic compounds, all of which can diminish due to ripening, poor storage, or enzymatic activity (Aslam et al. 2026). Despite providing essential nutrients and antioxidants, fruits and vegetables often suffer postharvest losses caused by physical damage, inadequate storage, or cold sensitivity. Fruits usually have a higher total soluble solids content during ripening and postharvest storage because moisture content decreases rapidly due to metabolism and respiration, and free sugar accumulation increases. A study also observed a similar increase in TSS in arils treated with essential oil from lemongrass and flaxseed gum. *Mentha piperita* L. essential oil and increasing the amounts of guar gum and essential oil help to maintain TSS values by creating a modified atmosphere, reducing the respiration rate, and possibly affecting the fruit's metabolic processes through the action of the essential oil (Seyedi et al. 2026).

Organic acids in fruits are the main source of TA (Bi et al. 2026). As these organic acids are broken down through respiration, TA typically declines gradually throughout storage (Ozturk et al. 2026). This reduction occurs because organic acids are utilized as energy substrates for fruit ripening and senescence, supporting metabolic processes that complete the tricarboxylic acid cycle (Li, Li, et al. 2026).

#### 3.2.4 pH

The pH values of the mango samples increased slightly during storage, indicating a shift toward more neutral conditions; however, all treatments showed an overall increasing trend. While treatment with 100% mixed flower honey maintained the pH at a relatively stable level, thereby indicating delayed spoilage and improved preservation of the fruit's internal quality, the pH in samples coated with *Aloe vera* and mixed flower honey increased by 1%–2% on Day 20 (Table 4). For pH, the two‐way ANOVA revealed that treatment (*F*_5,60_ = 13.73, *p* < 0.0001), storage time (*F*_4,60_ = 19.63, *p* < 0.0001), and their interaction (*F*_20,60_ = 4.182, *p* < 0.0001) significantly influenced pH (Table S5).

**TABLE 4:** Effect of *Aloe vera* and mixed flower honey coating on pH values during refrigerated storage.

Treatment	Storage duration (Day)				
	D_0_	D_5_	D_10_	D_15_	D_20_
T_0_	^A^4.74 ± 0.03^ab^	^A^4.80 ± 0.01^ab^	^A^4.85 ± 0.04^ab^	^A^4.70 ± 0.02^b^	^A^4.80 ± 0.21^a^
T_1_	^B^4.78 ± 0.05^a^	^AB^4.91 ± 0.03^a^	^AB^4.92 ± 0.10^a^	^A^4.97 ± 0.04^a^	^AB^4.82 ± 0.05^a^
T_2_	^B^4.75 ± 0.04^ab^	^B^4.67 ± 0.05^b^	^B^4.77 ± 0.07^ab^	^A^4.99 ± 0.01^a^	^B^4.76 ± 0.07^a^
T_3_	^A^4.69 ± 0.02^b^	^A^4.71 ± 0.04^b^	^A^4.75 ± 0.03^b^	^A^4.73 ± 0.06^b^	^A^4.74 ± 0.02^a^
T_4_	^B^4.71 ± 0.01^b^	^B^4.73 ± 0.04^b^	^A^4.88 ± 0.04^ab^	^A^4.95 ± 0.03^a^	^AB^4.82 ± 0.05^a^
T_5_	^B^4.72 ± 0.10^ab^	^B^4.71 ± 0.01^b^	^AB^4.80 ± 0.01^ab^	^A^4.89 ± 0.01^ab^	^B^4.77 ± 0.02^a^

*Note:* a–c means followed by different superscript alphabets in each column are significantly different (*p* < 0.05) among different storage durations within the same temperature. A–D means followed by different superscript alphabets in each row are significantly different (*p* < 0.05) among different treatments. Values are mean ± SD (*n* = 3). Different superscript letters indicate significant differences among treatments within the same storage time (*p* < 0.05) according to DMRT. Two‐way ANOVA showed significant effects of treatment (T), time (D), and their interaction (T × D) at *p* < 0.05, unless otherwise noted. T_0_ is our control untreated mango, T_1_ is mango coated with 100% *Aloe vera*, T_2_ is mango coated with 100% mixed flower honey, T_3_ is mango coated with 50% mixed flower honey and 50% *Aloe vera*, T_4_ is mango coated with 25% mixed flower honey and 75% *Aloe vera*, and T_5_ is mango coated with 75% mixed flower honey and 25% *Aloe vera* .

During storage, pH levels increased slightly overall, indicating a rise in alkalinity, yet treatment with *Aloe vera* and mixed flower honey helped maintain greater pH stability than the other samples (Balkrishna et al. 2026). All samples exhibited a consistent decline in titratable acidity (*p* ≤ 0.05), with the treated fruit cubes showing a 65% decrease by the 20th day, and the TSS/TA ratio increased during storage such that higher ratios in the treated fruits reflected improved taste quality through a better balance between sweetness and acidity.

Maintaining fruit quality during storage requires keeping pH at an optimal level, since acidity strongly influences flavor perception. In fruits, the balance between sugars and organic acids determines the perceived sweetness–sourness ratio, which is a key driver of overall flavor quality and consumer acceptability (Aslam et al. 2026). By moderating the decline in TA and the rise in TSS, the coatings help preserve a more favorable TSS/TA ratio, which is widely used as an index of eating quality and balanced flavor in mango and other climacteric fruits (Tang et al. 2026). As fruits ripen, a slight rise in acidity typically occurs, which could explain the variations in pH observed between the coated and uncoated fruits throughout the storage time.

Similar results were reported by Iqbal et al. (2026), who studied the effect of a multilayered antimicrobial biodegradable edible coating made from hydroxypropylmethylcellulose (HPMC) and chitosan on the storage of fresh‐cut mango (Iqbal et al. 2026). The biodegradable coating effectively delayed compositional changes in fresh‐cut mango. Biochemical reactions between the fruit and the coating material, along with slower respiration and metabolic activity, are common reasons for changes in pH (Çolak et al. 2025). In addition, the increase in pH may result from the breakdown of acids associated with respiration during storage (Alan et al. 2025).

#### 3.2.5 Antioxidant Content (Vitamin C, Total Phenol Content (TPC), and Total Flavonoid Content (TFC))

Figure 4A represents the influence of the *Aloe vera* and mixed flower honey coating technique on the vitamin C concentration of the fresh‐cut mango under storage conditions. Vitamin C concentration decreased significantly during storage, as confirmed by the significant effects of treatment, storage time, and their interaction. There was a minor reduction in vitamin C concentration at Days 5, 10, 15, and 20 (approximately 1.49%–1.65%) compared with the control group (3.07%) (Figure 4A) where the control sample (T_0_) lost approximately 23.8 mg 100/g of vitamin C by Day 20, the two‐way ANOVA revealed that treatment (*F*_5,60_ = 42.58, *p* < 0.0001), storage time (*F*_4,60_ = 265.2, *p* < 0.0001), and their interaction (*F*_20,60_ = 6.913, *p* < 0.0001) significantly influenced Vitamin C (Table S6), whereas At Day 20, the control (T_0_) showed the greatest vitamin C loss (approximately 23.8 mg/100 g), whereas 100% *Aloe vera* (T_1_) showed the lowest loss (7.91 mg/100 g), confirming that *Aloe vera* significantly retarded vitamin C degradation. This protective effect is consistent with previous reports that *Aloe vera* –based edible coatings reduce oxygen permeability and oxidative enzyme activity, thereby limiting ascorbic acid oxidation in fresh‐cut fruit cubes. In this study, the vitamin C content of fresh mango was found to be 31.30 mg/100 g, which was slightly higher than that of Abida et al. (2026), who found 18 mg/100 g of vitamin C in fresh mango (Abida et al. 2026). It was observed that the loss of vitamin C was 23.8 and 7.91 mg/100 g in the control sample and 100% *Aloe vera‐coated* sample, respectively. All coating formulations helped retain vitamin C, but the higher concentration of *Aloe vera* gel solution was more effective at reducing vitamin C loss in fresh‐cut mango. Saha et al. (2023) and Roy et al. (2023) supported this finding, reporting that *Aloe vera* gel coating had low oxygen permeability, which reduced enzyme activity and prevented oxidation of ascorbic acid (Roy et al. 2023; Saha et al. 2023). Shivaswamy et al. (2026) found similar results in fresh‐cut mango, where vitamin C content decreased significantly during storage (Shivaswamy et al. 2026).

**FIGURE 4A:**
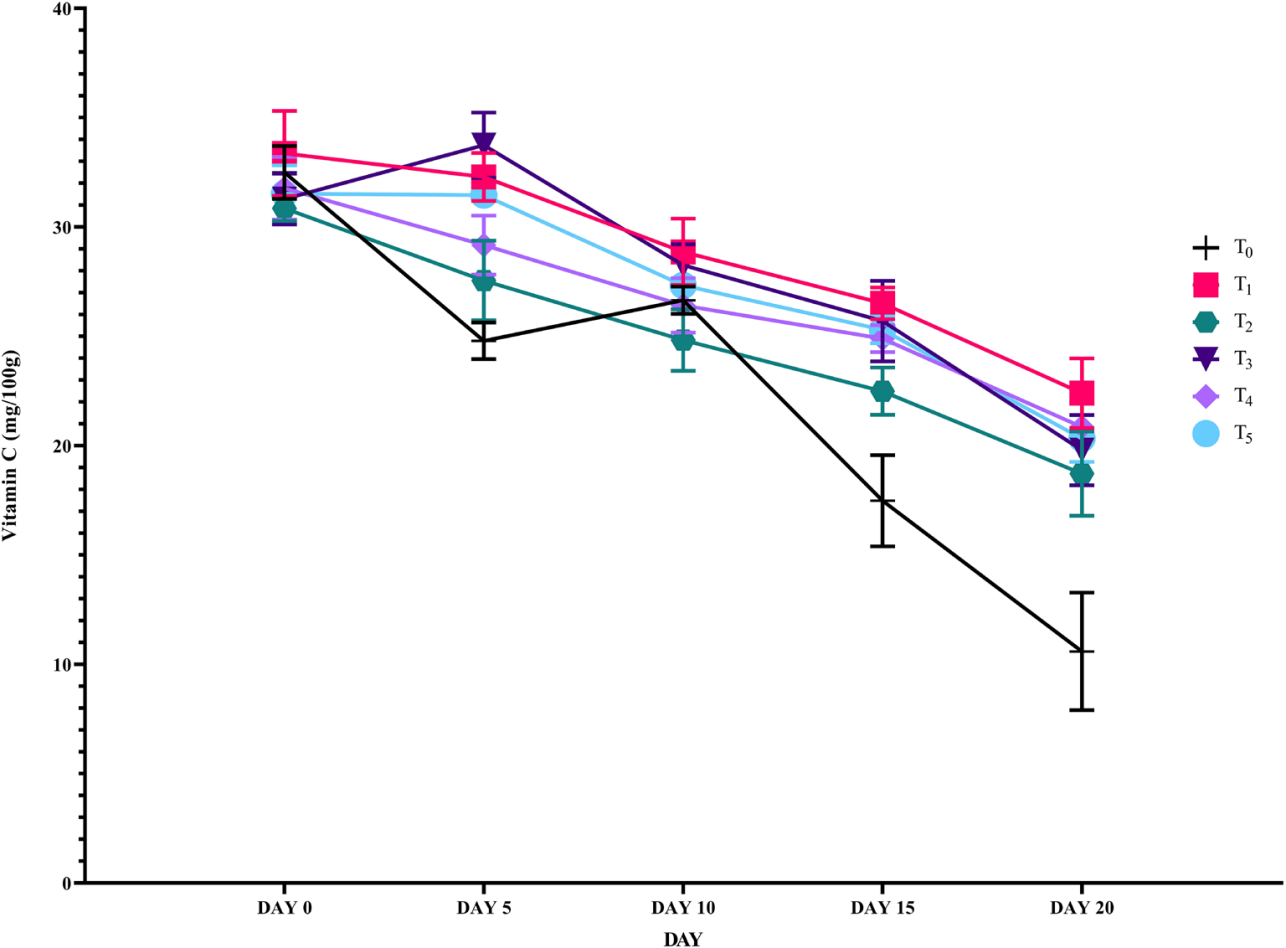
Effect of *Aloe vera* and mixed flower honey coating on Vitamin C during refrigerated storage. Values are mean ± SD (*n* = 3). Two‐way ANOVA showed significant effects of treatment (T), time (D), and their interaction (T × D) at *p* < 0.05, unless otherwise noted. T_0_ is our control untreated mango, T_1_ is mango coated with 100% *Aloe vera*, T_2_ is mango coated with 100% mixed flower honey, T_3_ is mango coated with 50% mixed flower honey and 50% *Aloe vera*, T_4_ is mango coated with 25% mixed flower honey and 75% *Aloe vera*, and T_5_ is mango coated with 75% mixed flower honey and 25% *Aloe vera* .

In coated samples, both phenol and flavonoid contents decreased during storage time. From the 5th day of storage, using the *Aloe vera* and mixed flower honey's different coating treatments retained the phenolic content by 6.60%, 8.53%, 10.51%, 16.23%, 11.16% and 10.77%, respectively while on 20th day of storage time, using the treatments significantly (*p* ≤ 0.05) decreased the phenolic content by 51.68%, 27.10%, 22.51%, 32.09%, 31.72% and 27.18%, respectively. The highest decrease was observed for treatment T_0_ (control), at 51.68%, while the lowest was observed for treatment T_2_ (100% mixed flower honey), at 22.51% (Figure 4B). The two‐way ANOVA revealed that treatment (*F*_5,60_ = 133.3, *p* < 0.0001), storage time (*F*_4,60_ = 1997, *p* < 0.0001), and their interaction (*F*_20,60_ = 54.53, *p* < 0.0001) significantly influenced TPC (Table S7). Total phenol is also an important factor for the maturity index. Figure 4B shows the changes in total phenol throughout the storage periods. The results showed that total phenol content in fresh mango samples ranged from 90.78 to 99.15 mg GAE per 100 g. During storage, total phenol content decreased to between 98.39 and 47.54 mg GAE per 100 g for the control and between 96.76 and 74.98 mg GAE per 100 g for the 100% mixed flower honey‐coated sample. The loss in the control sample was 50.85, and in the coated samples was 24.86, 21.78, 30.24, 29.50, and 25.95 mg GAE per 100 g, respectively. This result is similar to that of Kumar et al. (2026) and Abida et al. (2026), who reported that it could be 33.01 to 146.48 mg GAE per 100 g, depending on the varieties, environment, plant age, soil condition, rainfall, temperature, relative humidity, wind, and even sunshine (Abida et al. 2026; Kumar et al. 2026). According to Roy et al. (2023) and Saha et al. (2023), lowering total phenol content can reduce astringency during storage (Roy et al. 2023; Saha et al. 2023). More monomeric components can convert to polymeric components, which also reduces total phenol content. This study shows that the loss of total phenols is reduced by the application of *Aloe vera* and honey coating, as also shown by Akkad et al. (2026) and Main et al. (2026).

**FIGURE 4B:**
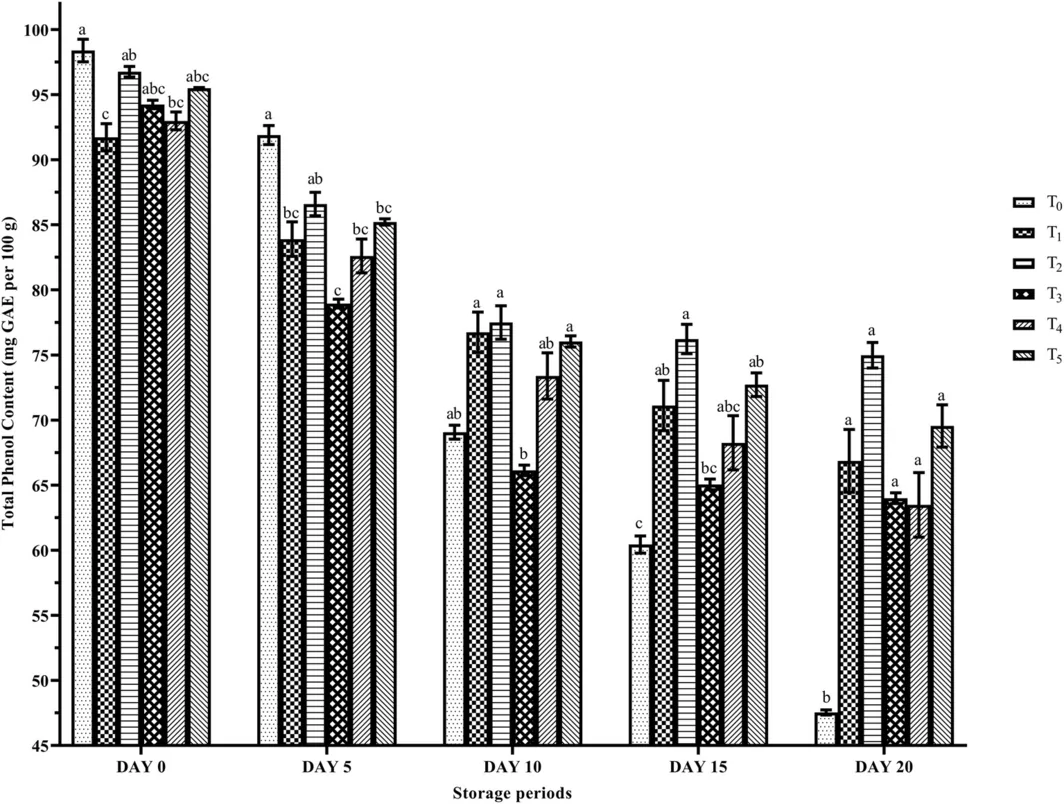
Effect of *Aloe vera* and mixed flower honey coating on TPC during refrigerated storage. Values are mean ± SD (*n* = 3). Different superscript letters indicate significant differences among treatments within the same storage time (*p* < 0.05) according to DMRT. Two‐way ANOVA showed significant effects of treatment (T), time (D), and their interaction (T × D) at *p* < 0.05, unless otherwise noted. T_0_ is our control untreated mango, T_1_ is mango coated with 100% *Aloe vera*, T_2_ is mango coated with 100% mixed flower honey, T_3_ is mango coated with 50% mixed flower honey and 50% *Aloe vera*, T_4_ is mango coated with 25% mixed flower honey and 75% *Aloe vera*, and T_5_ is mango coated with 75% mixed flower honey and 25% *Aloe vera* .

On Day 20, flavonoids decreased by more than 50% in untreated samples. Total flavonoid content decreased significantly during storage in all treatments (Figure 4C). From Day 0 to 20, TFC decreased by 51.67%, 27.10%, 22.51%, 32.11%, 31.73%, and 27.16% in T_0_, T_1_, T_2_, T_3_, T_4_, and T_5_, respectively. The greatest flavonoid loss occurred in the uncoated control (T_0_), whereas T_2_ (100% mixed flower honey) showed the lowest loss and the highest TFC retention among the treatments (Figure 4C). The two‐way ANOVA revealed that treatment (*F*_5,60_ = 172.2, *p* < 0.0001), storage time (*F*_4,60_ = 4076, *p* < 0.0001), and their interaction (*F*_20,60_ = 134.5, *p* < 0.0001) significantly influenced TFC (Table S8).

**FIGURE 4C:**
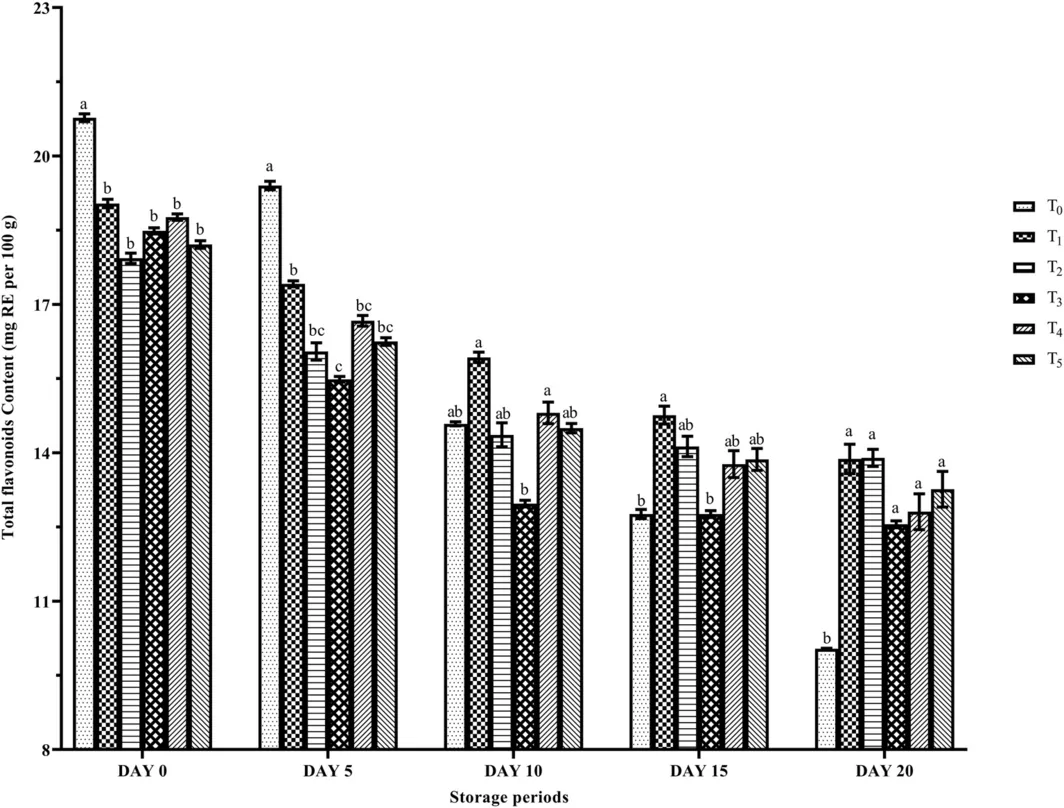
Effect of *Aloe vera* and mixed flower honey coating on TFC during refrigerated storage. Values are mean ± SD (*n* = 3). Different superscript letters indicate significant differences among treatments within the same storage time (*p* < 0.05) according to DMRT. Two‐way ANOVA showed significant effects of treatment (T), time (D), and their interaction (T × D) at *p* < 0.05, unless otherwise noted. T_0_ is our control untreated mango, T_1_ is mango coated with 100% *Aloe vera*, T_2_ is mango coated with 100% mixed flower honey, T_3_ is mango coated with 50% mixed flower honey and 50% *Aloe vera*, T_4_ is mango coated with 25% mixed flower honey and 75% *Aloe vera*, and T_5_ is mango coated with 75% mixed flower honey and 25% *Aloe vera* .

Phenolic and flavonoid compounds contribute to mango's antioxidant capacity by scavenging reactive oxygen species and limiting oxidative damage during storage (Sati et al. 2026). The greater retention of vitamin C, TPC, and TFC in coated mangoes suggests that the coatings reduced oxidative degradation, likely by limiting oxygen transfer and slowing metabolic activity (Kuldeep et al. 2025).

Non‐enzymatic antioxidants, such as phenolic and flavonoid compounds, are essential for strengthening fruits' antioxidant defenses against oxidative stress during ripening. By scavenging free radicals and preventing hydrogen peroxide from generating additional harmful radicals, these bioactive compounds help maintain fruit quality. Essential oils extracted from medicinal plants using different techniques are known to increase the activity of the enzyme phenylalanine ammonia‐lyase (PAL), which in turn promotes phenolic compound synthesis. Additionally, essential oils themselves possess antioxidant properties mainly due to their phenolic and flavonoid content. Previous studies reported that applying *Aloe vera* to pineapples and oranges slows ascorbic acid loss and inhibits its oxidation by limiting oxygen penetration into the fruit. However, high gel concentrations significantly decreased ascorbic acid content, probably because elevated internal CO_2_ levels within the fruit promote its breakdown.

#### 3.2.6 Color

Color is an important quality parameter for fresh‐cut mango. In this study, we assessed the color of coated and uncoated fresh‐cut mangoes by measuring lightness (L*), yellowness (a*), redness (b*), hue angle (*H*), and total color difference (∆*E*) over 20 days of storage at 4°C. Table 5 shows the changes in the color of fresh‐cut mangoes during storage. According to Table 5, changes in L*, a*, b*, hue angle, and ΔE were significant (*p* < 0.05). At Day 20, uncoated mango (T_0_) had significantly lower L*, b*, hue angle, and ΔE, and significantly higher a* than the 100% *Aloe vera* coating (T_1_).

**TABLE 5:** Effect of *Aloe vera* and mixed flower honey coating on color during refrigerated storage.

Treatment	Storage duration (Day)				
	D_0_	D_5_	D_10_	D_15_	D_20_
L* value					
T_0_	^A^49.17 ± 3.59^b^	^AB^43.37 ± 6.71^a^	^AB^44.97 ± 5.15^ab^	^AB^45.51 ± 1.70^a^	^B^38.40 ± 8.02^b^
T_1_	^A^55.13 ± 1.06^a^	^AB^51.03 ± 2.14^a^	^B^41.00 ± 1.56^b^	^AB^48.12 ± 7.61^a^	^AB^52.98 ± 6.47^a^
T_2_	^A^50.22 ± 2.26^ab^	^A^49.80 ± 4.90^a^	^A^46.80 ± 6.46^ab^	^A^42.88 ± 0.77^a^	^A^49.92 ± 2.89^a^
T_3_	^A^55.20 ± 3.49^a^	^B^42.51 ± 3.44^a^	^AB^50.04 ± 5.11^a^	^AB^46.60 ± 2.89^a^	^AB^47.53 ± 1.55^ab^
T_4_	^A^52.52 ± 0.79^ab^	^B^43.48 ± 3.13^a^	^AB^49.03 ± 1.89^ab^	^AB^49.83 ± 5.16^a^	^AB^48.86 ± 2.48^ab^
T_5_	^A^52.65 ± 2.18^ab^	^B^45.02 ± 3.83^a^	^AB^48.62 ± 4.49^ab^	^AB^46.44 ± 2.94^a^	^AB^48.77 ± 2.31^ab^
a* value					
T_0_	^AB^12.17 ± 0.15^a^	^AB^12.32 ± 0.13^a^	^B^12.03 ± 0.20^a^	^AB^12.47 ± 0.08^a^	^A^12.61 ± 0.34^a^
T_1_	^A^11.84 ± 0.31^a^	^AB^11.60 ± 0.17^b^	^A^11.77 ± 0.46^ab^	^B^11.07 ± 0.09^b^	^B^11.16 ± 0.10^b^
T_2_	^A^11.95 ± 0.55^a^	^A^11.75 ± 0.31^ab^	^A^11.58 ± 0.23^abc^	^A^11.28 ± 0.21^b^	^A^11.58 ± 0.46^b^
T_3_	^A^11.89 ± 0.62^a^	^AB^11.59 ± 0.25^b^	^B^11.21 ± 0.16^c^	^AB^11.34 ± 0.29^b^	^B^11.12 ± 0.18^b^
T_4_	^AB^11.70 ± 0.12^a^	^A^11.90 ± 0.42^ab^	^BC^11.27 ± 0.20^bc^	^C^11.10 ± 0.26^b^	^BC^11.23 ± 0.14^b^
T_5_	^A^11.85 ± 0.43^a^	^AB^11.75 ± 0.15^ab^	^BC^11.35 ± 0.20^bc^	^C^11.24 ± 0.25^b^	^BC^11.31 ± 0.12^b^
b* value					
T_0_	^AB^31.77 ± 0.96^a^	^AB^31.22 ± 1.44^ab^	^AB^32.15 ± 5.31^a^	^A^33.39 ± 3.96^a^	^B^24.01 ± 5.19^b^
T_1_	^AB^32.87 ± 0.88^a^	^AB^29.41 ± 2.75^ab^	^B^28.85 ± 0.79^a^	^A^33.94 ± 0.82^a^	^A^34.04 ± 1.89^a^
T_2_	^A^34.42 ± 2.10^a^	^A^27.72 ± 5.14^b^	^A^26.65 ± 0.50^a^	^A^30.04 ± 5.28^a^	^A^29.84 ± 0.83^ab^
T_3_	^A^33.11 ± 2.26^a^	^A^29.57 ± 0.13^ab^	^A^29.36 ± 1.48^a^	^A^30.00 ± 4.86^a^	^A^30.98 ± 3.71^ab^
T_4_	^AB^32.76 ± 0.43^a^	^A^35.69 ± 2.85^a^	^AB^32.00 ± 1.13^a^	^B^29.91 ± 2.36^a^	^B^30.44 ± 1.72^ab^
T_5_	^A^33.43 ± 1.60^a^	^A^30.99 ± 2.71^ab^	^A^29.34 ± 1.04^a^	^A^29.98 ± 4.17^a^	^A^30.42 ± 2.09^ab^
**Hue angle**					
T_0_	^A^1.20 ± 0.01^b^	^A^1.19 ± 0.02^ab^	^A^1.21 ± 0.05^ab^	^A^1.21 ± 0.04^a^	^B^1.08 ± 0.08^b^
T_1_	^AB^1.23 ± 0.01^a^	^B^1.19 ± 0.04^ab^	^B^1.19 ± 0.01^ab^	^A^1.26 ± 0.01^a^	^A^1.26 ± 0.02^a^
T_2_	^A^1.24 ± 0.01^a^	^A^1.16 ± 0.08^b^	^A^1.16 ± 0.01^b^	^A^1.21 ± 0.06^a^	^A^1.20 ± 0.02^a^
T_3_	^A^1.23 ± 0.01^a^	^A^1.20 ± 0.01^ab^	^A^1.21 ± 0.02^ab^	^A^1.21 ± 0.05^a^	^A^1.23 ± 0.04^a^
T_4_	^A^1.23 ± 0.01^a^	^A^1.25 ± 0.04^a^	^A^1.23 ± 0.01^a^	^A^1.21 ± 0.02^a^	^A^1.22 ± 0.02^a^
T_5_	^A^1.23 ± 0.01^a^	^A^1.21 ± 0.04^ab^	^A^1.20 ± 0.01^ab^	^A^1.21 ± 0.04^a^	^A^1.22 ± 0.03^a^
**∆*E***					
T_0_	^A^59.84 ± 2.41^a^	^AB^54.90 ± 6.15^a^	^AB^56.59 ± 7.15^a^	^AB^57.83 ± 3.64^a^	^B^47.05 ± 9.29^b^
T_1_	^A^65.27 ± 1.40^a^	^AB^60.04 ± 3.15^a^	^B^51.50 ± 1.79^a^	^AB^60.00 ± 6.58^a^	^A^63.98 ± 6.38^a^
T_2_	^A^62.05 ± 3.10^a^	^A^59.62 ± 6.61^a^	^A^55.14 ± 5.77^a^	^A^53.66 ± 3.61^a^	^A^59.31 ± 2.77^ab^
T_3_	^A^65.46 ± 4.20^a^	^B^53.09 ± 2.88^a^	^AB^59.63 ± 5.09^a^	^AB^56.61 ± 5.01^a^	^AB^57.85 ± 3.29^ab^
T_4_	^A^62.99 ± 0.91^a^	^A^57.51 ± 4.05^a^	^A^59.63 ± 2.20^a^	^A^59.17 ± 5.59^a^	^A^58.65 ± 2.99^ab^
T_5_	^A^63.48 ± 2.73^a^	^A^55.91 ± 4.55^a^	^A^57.93 ± 4.33^a^	^A^56.43 ± 4.58^a^	^A^58.59 ± 3.00^ab^

*Note:* a–c means followed by different superscript alphabets in each column are significantly different (*p* < 0.05) among different storage durations within the same temperature. A–D means followed by different superscript alphabets in each row are significantly different (*p* < 0.05) among different treatments. Values are mean ± SD (*n* = 3). Different superscript letters indicate significant differences among treatments within the same storage time (*p* < 0.05) according to DMRT. Two‐way ANOVA showed significant effects of treatment (T), time (D), and their interaction (T × D) at *p* < 0.05, unless otherwise noted. T_0_ is our control untreated mango, T_1_ is mango coated with 100% *Aloe vera*, T_2_ is mango coated with 100% mixed flower honey, T_3_ is mango coated with 50% mixed flower honey and 50% *Aloe vera*, T_4_ is mango coated with 25% mixed flower honey and 75% *Aloe vera*, and T_5_ is mango coated with 75% mixed flower honey and 25% *Aloe vera* .

The two‐way ANOVA revealed that treatment (*F*_5,60_ = 3.102, *p* = 0.0148), storage time (*F*_4,60_ = 7.757, *p* < 0.0001), and their interaction (*F*_20,60_ = 1.892, *p* = 0.0301) significantly influenced L* (Table S9). The two‐way ANOVA revealed that treatment (*F*_5,60_ = 21.15, *p* < 0.0001), storage time (*F*_4,60_ = 9.683, *p* < 0.0001), and their interaction (*F*_20,60_ = 1.880, *p* = 0.0314) significantly influenced a* (Table S10). The two‐way ANOVA revealed that storage time (*F*_4,60_ = 3.927, *p* = 0.0067) and the interaction between treatment and storage time (*F*_20,60_ = 2.119, *p* = 0.0133) significantly influenced b*, whereas treatment did not significantly influence b* (*F*_5,60_ = 1.499, *p* = 0.2036) (Table S11). The two‐way ANOVA revealed that treatment (*F*_5,60_ = 5.102, *p* = 0.0006), storage time (*F*_4,60_ = 2.851, *p* = 0.0313), and their interaction (*F*_20,60_ = 3.000, *p* = 0.0005) significantly influenced hue angle (Table S12). The two‐way ANOVA revealed that storage time significantly influenced ∆*E* (*F*_4,60_ = 6.727, *p* = 0.0002), whereas treatment (*F*_5,60_ = 2.156, *p* = 0.0710) and the interaction between treatment and storage time (*F*_20,60_ = 1.464, *p* = 0.1294) did not significantly influence ∆*E* (Table S13).

However, after storage, fresh‐cut mango coated with 100% *Aloe vera* showed a higher L* value than all other coated and uncoated mango samples. According to Table 5, a* increased in uncoated mangoes during storage, while a* declined in all coated mangoes over the same period. In addition, the values of b* for coated samples were relatively stable compared to those of uncoated samples over 20 days of storage. At the end of the storage period, the b* value of 100% *Aloe vera* coating was nearly similar to that on the initial day, as observed in a similar result (Abida et al. 2026). However, small changes in a* and b* values are a good indication of the absence of oxidative browning of fresh pulp (Sharma et al. 2026; Xie et al. 2026). In the control sample, hue angle decreased over time, while it increased in the coated sample. This increasing trend in hue angle in the coated sample indicated less redness and more yellowness, which is also reported with increasing hue angle (Abida et al. 2026; Sharma et al. 2026).

#### 3.2.7 Microbiological Analysis

The impact of *Aloe vera* and mixed flower honey coatings on microbial growth in fresh‐cut mangoes is presented in Table 6. Total microbial load, including molds and yeasts, decreased significantly (*p* < 0.05) during storage, with the lowest counts consistently in T_1_ (100% *Aloe vera* coating), followed by the other coated treatments. The two‐way ANOVA revealed that treatment (*F*_5,60_ = 8595, *p* < 0.0001), storage time (*F*_4,60_ = 11,194, *p* < 0.0001), and their interaction (*F*_20,60_ = 7497, *p* < 0.0001) significantly influenced TVC (Table S14). The two‐way ANOVA revealed that treatment (*F*_5,60_ = 963.9, *p* < 0.0001), storage time (*F*_4,60_ = 5051, *p* < 0.0001), and their interaction (*F*_20,60_ = 525.1, *p* < 0.0001) significantly influenced TMYC (Table S15). In contrast, the control samples showed the highest microbial counts throughout storage. These findings are consistent with previous studies showing that *Aloe vera* ‐ and honey‐based coatings can effectively inhibit microbial growth in fresh‐cut fruit cubes. The control group had the highest total microbial counts, as well as the highest mold and yeast counts, while the different coating treatment levels had the lowest counts. In addition, the microbial load on the control mango sample increased markedly from 1.79 × 10^3^ to 5.27 × 10^6^ CFU/g after 20 days of storage, while all coating compositions prepared at different levels of *Aloe vera* and mixed flower honey inhibited microbial growth throughout the storage period. These findings are consistent with the studies by Roy et al. (2023) and Saha et al. (2023), who found that coatings based on *Aloe vera* and honey can effectively inhibit microbial growth on fresh‐cut papaya and fresh‐cut guava, respectively (Roy et al. 2023; Saha et al. 2023). In addition, Nagaraja et al. (2026), Bin et al. (2026), and You et al. (2026) reported that lower temperatures could retard microbial growth, thus lowering the respiration rate and providing a longer shelf‐life for fresh‐cut products (Bin et al. 2026; Nagaraja et al. 2026; You et al. 2026).

**TABLE 6:** Effect of *Aloe vera* and mixed flower honey coating on microbial growth during refrigerated storage.

Treatment	Microbial growth (×10^3^ CFU/g)					Total mold and yeast counts (×10^1^ CFU/g)				
	D_0_	D_5_	D_10_	D_15_	D_20_	D_0_	D_5_	D_10_	D_15_	D_20_
T_0_	1.79 ± 45.09^b^	3.23 ± 72.34^b^	42.00 ± 10.00^b^	470.00 ± 45.82^a^	5270.00 ± 9.84^a^	0	0	5.00 ± 1.00^a^	23.70 ± 1.53^a^	92.33 ± 0.60^a^
T_1_	2.02 ± 100.66^b^	1.53 ± 65.57^b^	21.46 ± 60.77^b^	23.93 ± 11.37^b^	26.30 ± 13.52^b^	0	0	0^b^	5.00 ± 1.00^b^	33.00 ± 1.00^b^
T_2_	2.04 ± 45.09^b^	2.10 ± 100.66^b^	25.37 ± 24.54^b^	26.16 ± 10.11^b^	277.00 ± 62.44^b^	0	0	0^b^	7.00 ± 1.00^b^	15.67 ± 2.52^b^
T_3_	1.87 ± 57.74^b^	2.71 ± 70.95^b^	206.67 ± 57.73^a^	26.93 ± 40.4^b^	350.33 ± 25.16^b^	0	0	0^b^	3.33 ± 0.58^b^	17.33 ± 2.52^b^
T_4_	2.20 ± 45.09^b^	16.97 ± 450.92^a^	236.67 ± 58.59^a^	249.00 ± 26.45^ab^	261.00 ± 26.45^b^	0	0	0^b^	6.33 ± 0.58^b^	17.67 ± 1.53^b^
T_5_	7.57 ± 321.46^a^	8.57 ± 493.29^b^	159.00 ± 21.21^a^	170.33 ± 15.04^ab^	272.00 ± 6.24^b^	0	0	0^b^	3.67 ± 0.58^b^	46.67 ± 2.08^b^

*Note:* Values are mean ± SD (*n* = 3). Different superscript letters indicate significant differences among treatments within the same storage time (*p* < 0.05) according to DMRT. Two‐way ANOVA showed significant effects of treatment (T), time (D), and their interaction (T × D) at *p* < 0.05, unless otherwise noted. Detection limits: Total aerobic bacteria = 0.1 × 10^3^ CFU/g (equivalent to 1.0 × 10^2^ CFU/g, derived from 0.1 mL of the 10^−1^ dilution); Molds & yeasts = 1.0 × 10^1^ CFU/g (derived from 1.0 mL of the 10^−1^ dilution). T_0_ is our control untreated mango, T_1_ is mango coated with 100% *Aloe vera*, T_2_ is mango coated with 100% mixed flower honey, T_3_ is mango coated with 50% mixed flower honey and 50% *Aloe vera*, T_4_ is mango coated with 25% mixed flower honey and 75% *Aloe vera*, and T_5_ is mango coated with 75% mixed flower honey and 25% *Aloe vera* .

#### 3.2.8 Storage Studies of Mango

Over time, the percentage of spoiled fruits increased, particularly in those without any coating. By the end of the experiment, fruit cubes treated with 100% mixed flower honey and combined honey showed the least spoilage, demonstrating their ability to provide better protection. Overall, coated fruit cubes had lower spoilage rates than the control because the edible coatings prevented fruit deterioration. With 100% mixed flower honey, the spoilage rates for fruit cubes were noticeably low by the twentieth day of storage. We evaluated the organoleptic qualities of all mangoes during storage at refrigeration temperature (4°C). The evaluation covered color, taste, texture, and visible fungal infection, as shown in Table 7 below. For the whole bunch, these qualities did not change until 5 days into the period, after which changes began slowly. After 15 days, the color darkened from yellowish to brownish, and slight visible fungal infection appeared. On the twentieth day, all organoleptic qualities had changed significantly, making the mango unfit for consumption due to fungal infection. For the coated samples, the only difference noticed was softness until the 10th day, indicating that the coating materials provided better initial protection. The mixed‐coated samples were not accepted from the 10th day onward because their quality changed considerably. However, the mixed‐coated samples were better than the other samples and were accepted up to the 15th day, though by the 25th day all the samples were spoiled. As Álvarez‐Suárez et al. (2026) and Ye et al. (2026) concluded, if the sample is treated correctly and stored in a cool, dry place, then it can be stored for several months (Álvarez‐Suárez et al. 2026; Ye et al. 2026). Similar studies have shown that a mix of *Aloe vera* and honey (75:25) extends shelf life by 21 days (Fabien 2026).

**TABLE 7:** Sensory studies of coated mango.

Storage period	Treatment	Color	Flavor	Texture	Visual fungal growth	Remarks
Day 0	T_0_	Yellowish‐gold	Good	Hard	No growth	Good
	T_1_	Yellowish‐gold (Glossy)	Good	Hard	No growth	Good
	T_2_	Yellowish‐gold (Glossy)	Good	Hard	No growth	Good
	T_3_	Yellowish‐gold (Glossy)	Good	Hard	No growth	Good
	T_4_	Yellowish‐gold (Glossy)	Good	Hard	No growth	Good
	T_5_	Yellowish‐gold (Glossy)	Good	Hard	No growth	Good
Day 5	T_0_	Slightly Yellowish	Good	Hard	No growth	Good
	T_1_	Slightly Yellowish‐gold (Little Glossy)	Good	Hard	No growth	Good
	T_2_	Slightly Yellowish‐gold (Little Glossy)	Good	Hard	No growth	Good
	T_3_	Slightly Yellowish‐gold (Little Glossy)	Good	Hard	No growth	Good
	T_4_	Slightly Yellowish‐gold (Little Glossy)	Good	Hard	No growth	Good
	T_5_	Slightly Yellowish‐gold (Little Glossy)	Good	Hard	No growth	Good
Day 10	T_0_	Yellowish	Good	Semi Soft	No growth	Good
	T_1_	Slightly Yellowish	Good	Semi Soft	No growth	Good
	T_2_	Slightly Yellowish	Good	Semi Soft	No growth	Good
	T_3_	Slightly Yellowish	Good	Semi Soft	No growth	Good
	T_4_	Slightly Yellowish	Good	Semi Soft	No growth	Good
	T_5_	Slightly Yellowish	Good	Semi Soft	No growth	Good
Day 15	T_0_	Slightly Brownish	Slightly Off flavor	Soft	Highly growth	Spoiled
	T_1_	Yellowish	Good	Semi Soft	Slightly growth	Good
	T_2_	Yellowish	Good	Semi Soft	No growth	Good
	T_3_	Yellowish	Good	Semi Soft	No growth	Good
	T_4_	Yellowish	Good	Semi Soft	No growth	Good
	T_5_	Yellowish	Good	Semi Soft	No growth	Good
Day 20	T_0_	Dark Brown	Off flavor	No texture	Excessive growth	Spoiled
	T_1_	Brownish	Slightly Off flavor	Soft	Medium growth	Slightly Spoiled
	T_2_	Slightly Brownish	Slightly Off flavor	Soft	Medium growth	Slightly spoiled
	T_3_	Slightly Brownish	Slightly Off flavor	Semi Soft	Medium growth	Slightly spoiled
	T_4_	Slightly Brownish	Slightly Off flavor	Semi Soft	Medium growth	Slightly spoiled
	T_5_	Slightly Brownish	Slightly Off flavor	Semi Soft	Medium growth	Slightly spoiled
Day 25	T_0_	Fully Dark Brown	Off flavor	No texture	Excessive growth	Spoiled
	T_1_	Dark Brown	Off flavor	No texture	Excessive growth	Spoiled
	T_2_	Dark Brown	Off flavor	Semi Soft	Excessive growth	Spoiled
	T_3_	Dark Brown	Off flavor	Semi Soft	Excessive growth	Spoiled
	T_4_	Dark Brown	Off flavor	Semi Soft	Excessive growth	Spoiled
	T_5_	Dark Brown	Off flavor	Semi Soft	Excessive growth	Spoiled

#### 3.2.9 Correlation Analysis

Figure 5 shows the correlations between the 16 evaluated traits using Pearson correlation. At *p* ≤ 0.05, Pearson's correlation coefficient is proportional to the severity of the squares' size and color. Positive correlations are shown by red circles, whereas negative correlations are shown by blue circles. The correlation analysis revealed several significant positive and negative relationships. Among the observed correlations, the strong negative relationship between moisture loss and both firmness (−0.88) and antioxidant compounds such as total phenolic content (−0.85) and total flavonoid content (−0.91) emerges as the most important for understanding quality loss in fresh‐cut mango. This indicates that samples experiencing greater water loss also softened more rapidly and lost phenolics and flavonoids faster, consistent with the view that dehydration accelerates membrane damage, enzymatic browning, and oxidative degradation in cut fruit cubes. In contrast, treatments that limited moisture loss (notably T_1_ and T_4_) maintained higher firmness and antioxidant levels, likely contributing to slower sensory spoilage (Jama et al. 2026). These findings are consistent with reports on fresh‐cut pineapple, guava, mango, ber, and kiwifruit, in which lower water loss was associated with better firmness and antioxidant retention during cold storage (Nasrin et al. 2026; Parente et al. 2026; Roy et al. 2023; Saha et al. 2023; Seyedi et al. 2026).

**FIGURE 5:**
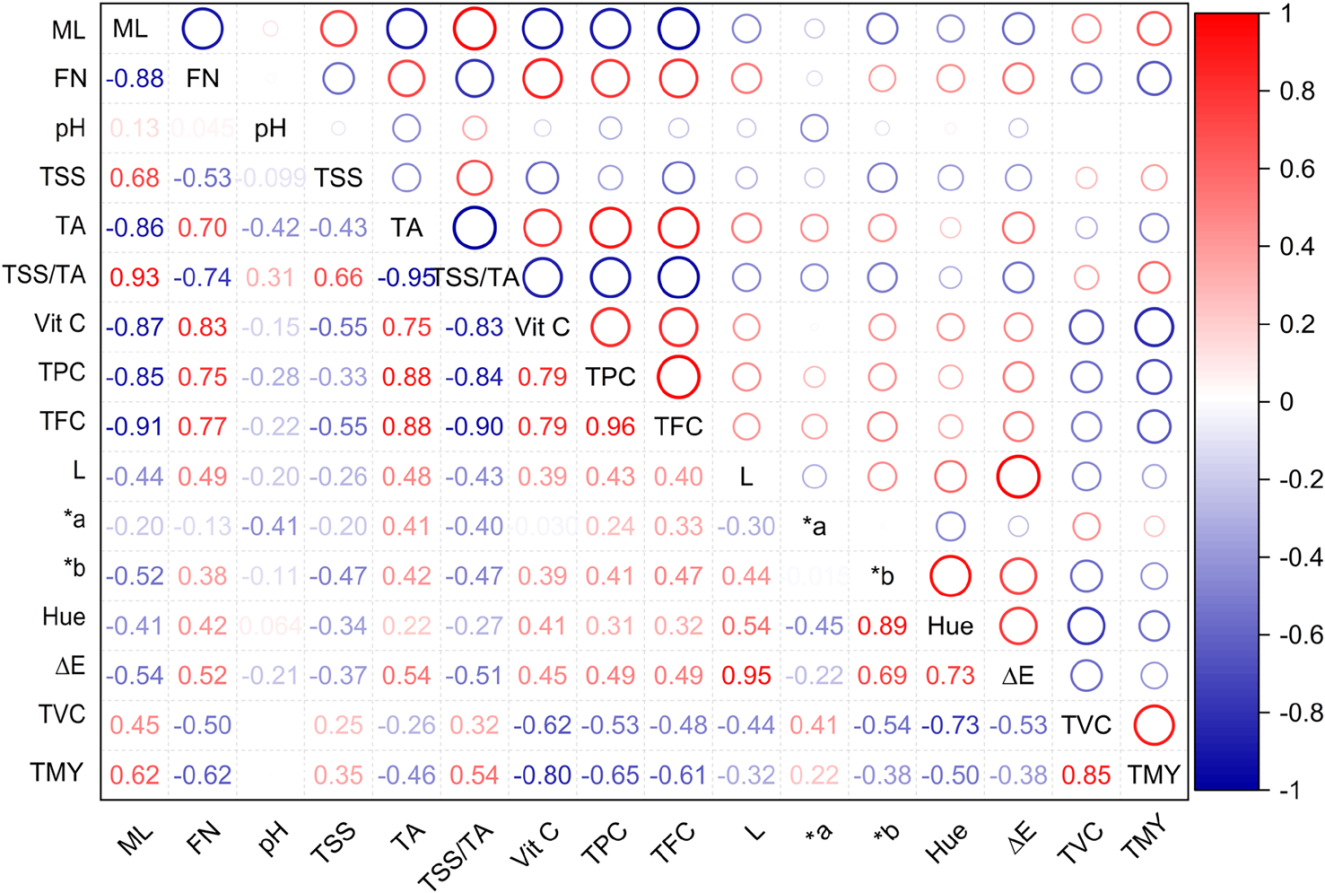
Pearson correlation analysis (*p* ≤ 0.05) of storage time and edible coating treatments with variable trait relationships, biochemical, microbial, and physiological parameters of mango. FN, firmness; ML, moisture loss; TA, titratable acidity; TFC, total flavonoid content; TMY, total mold and yeast count; TPC, total phenol content; TSS, total soluble solids; TSS/TA ratio, flavor index; TVC, total viable count; Vit C, vitamin C.

#### 3.2.10 Principal Component Analysis (PCA)

In Figure 6, PCA reveals treatment‐ and storage‐dependent variation in the biochemical and physiological traits of Mango. PCA illustrated the interrelationships among 16 biochemical and physiological traits across four storage periods and five coating treatments (T_1_–T_5_). The PCA further clarifies the key factors driving quality changes in coated fresh‐cut mango during storage. PC1, which accounted for 53.72% of the total variation, separated samples based on a quality gradient defined by firmness, vitamin C, and titratable acidity (positive loadings) versus moisture loss and TSS/TA (negative loadings). Treatments with higher *Aloe vera* content (particularly T_1_) clustered toward the positive side of PC1 at earlier storage times, indicating better retention of firmness, vitamin C, and acidity, and lower moisture loss. In contrast, the uncoated control and some mixed‐coating treatments shifted toward the negative side of PC1 at later storage days, reflecting advanced softening, greater water loss, and higher TSS/TA, all associated with over‐ripening and reduced consumer acceptability. PC2 (17.09% of variance) primarily captured storage duration, separating early (Days 0–5) from late (Days 15–20) storage samples. The movement of samples along PC2 over time, especially for the control, illustrates the progressive deterioration in quality attributes as storage advances. Together, the PCA results reinforce that moisture loss, firmness, vitamin C, and acidity are the key drivers discriminating high‐quality, well‐protected samples from those undergoing rapid senescence, and that the *Aloe vera* –based coatings effectively keep the fruit in the favorable region of this multivariate quality space for longer.

**FIGURE 6:**
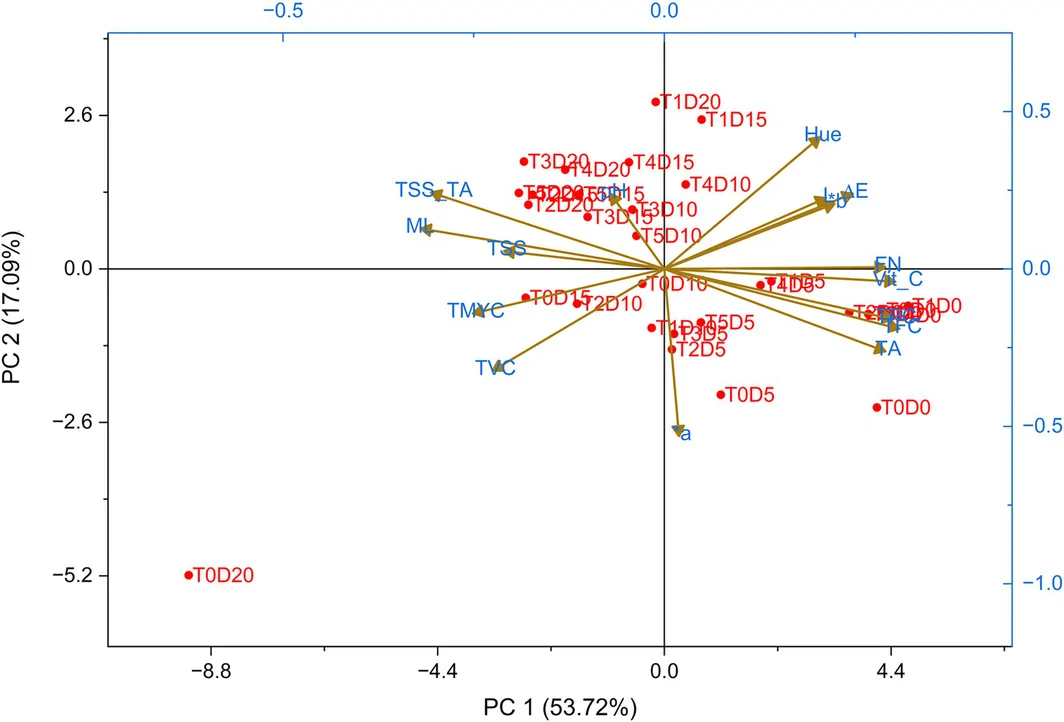
Principal component analysis (PCA) of storage time and edible coating treatments with variable trait relationships, biochemical, microbial, and physiological parameters of mango. FN, Firmness; ML, moisture loss; TA, titratable acidity; TFC, total flavonoid content; TMY, total mold and yeast count; TPC, total phenol content; TSS, total soluble solids; TSS/TA ratio, flavor index; TVC, total viable count; Vit C, vitamin C.

## 4 Conclusion

This research showed that mango quality can be preserved for 20 days of storage using *Aloe vera* and mixed flower honey in biodegradable edible coatings that act as protective barriers. This technique reduced deterioration and oxidation and improved retention of the fruit's nutritional value and physicochemical qualities during refrigeration. Among the applied coatings, the formulation consisting of 100% *Aloe vera* proved to be the most effective, as it significantly reduced moisture loss (by 48% relative to the uncoated control on Day 20), best maintained fruit firmness (retaining > 90%), exhibited the lowest microbial load (total aerobic bacteria, molds, and yeasts), and most effectively preserved key antioxidant compounds, including vitamin C, total phenolics, and flavonoids, while also delaying surface browning and spoilage throughout 20 days of refrigerated storage. This eco‐friendly approach is an excellent alternative to chemical preservatives, and these coatings have strong potential for large‐scale use to preserve climacteric fruits like mangoes, papayas, and guavas. Future research could examine the effects of these bio‐based coatings on other climacteric and non‐climacteric fruit varieties to expand knowledge. Utilizing state‐of‐the‐art techniques, such as encapsulating active compounds, could increase coating efficacy and improve performance. While the technique is promising, several issues need careful examination. These concerns relate to the higher costs of manufacturing and using these biodegradable films, especially when honey is added. Moreover, scaling up from lab experiments to industrial production becomes complicated because fruit species vary.

## 5 Limitations

This study used one local mango variety (Ashwina) and one refrigerated storage condition, so the results may not apply to other cultivars, harvest maturities, or temperatures. The work was laboratory‐scale and did not include commercial processing, packaging, transport, retail display, or cost analysis. We inferred the coating mechanism from changes in quality. We did not measure coating microstructure, thickness, gas or water‐vapor permeability. No trained sensory panel was used, so consumer liking was not quantified. Microbial tests were limited to total aerobic counts, molds, and yeasts, without identification of spoilage species.

## Author Contributions

**Tupura Khatun:** investigation, writing – original draft, data curation, formal analysis. **Md. Asaduzzaman:** conceptualization, methodology, validation, visualization, writing – review and editing, software, data curation, supervision, resources, project administration, funding acquisition.

## Funding

The authors have nothing to report.

## Conflicts of Interest

The authors declare no conflicts of interest.

## Supporting information


**Table S1:** Two‐way ANOVA results for moisture loss.
**Table S2:** Two‐way ANOVA results for Firmness.
**Table S3:** Two‐way ANOVA results for TSS.
**Table S4:** Two‐way ANOVA results for TSS/TA.
**Table S5:** Two‐way ANOVA results for pH.
**Table S6:** Two‐way ANOVA results for vitamin C.
**Table S7:** Two‐way ANOVA results for TPC.
**Table S8:** Two‐way ANOVA results for TFC.
**Table S9:** Two‐way ANOVA results for L*.
**Table S10:** Two‐way ANOVA results for a*.
**Table S11:** Two‐way ANOVA results for b*.
**Table S12:** Two‐way ANOVA results for Hue angle.
**Table S13:** Two‐way ANOVA results for ∆*E*.
**Table S14:** Two‐way ANOVA results for TVC.
**Table S15:** Two‐way ANOVA results for TMYC.

## Data Availability

The data that support the findings of this study are available on request from the corresponding author. The data are not publicly available due to privacy or ethical restrictions.
