# Supplementary material for: Aloe vera –Honey Edible Coating Prolongs Shelf Life and Preserves Quality of Refrigerated Fresh‐Cut Mango ( Mangifera indica )
Source: Food Sci Nutr. 2026 Oct 3;14(10):e72447. doi: 10.1002/fsn3.72447 (PMC13633574; doi:10.1002/fsn3.72447)
Supplement: Supplementary file 1 [file FSN3-14-e72447-s001.docx]

**Table S1 Two-way ANOVA results for moisture loss**

| Two-way ANOVA | Ordinary |  |  |  |  |
| --- | --- | --- | --- | --- | --- |
| Alpha | 0.05 |  |  |  |  |
|  |  |  |  |  |  |
| Source of Variation | % of total variation | P value | P value summary | Significant? |  |
| Interaction | 7.960 | <0.0001 | **** | Yes |  |
| Row Factor | 78.31 | <0.0001 | **** | Yes |  |
| Column Factor | 12.90 | <0.0001 | **** | Yes |  |
|  |  |  |  |  |  |
| ANOVA table | SS | DF | MS | F (DFn, DFd) | P value |
| Interaction | 488.6 | 20 | 24.43 | F (20, 60) = 28.86 | P<0.0001 |
| Row Factor | 4807 | 4 | 1202 | F (4, 60) = 1420 | P<0.0001 |
| Column Factor | 791.9 | 5 | 158.4 | F (5, 60) = 187.1 | P<0.0001 |
| Residual | 50.79 | 60 | 0.8466 |  |  |
|  |  |  |  |  |  |
| Data summary |  |  |  |  |  |
| Number of columns (Column Factor) | 6 |  |  |  |  |
| Number of rows (Row Factor) | 5 |  |  |  |  |
| Number of values | 90 |  |  |  |  |
|  |  |  |  |  |  |

**Table S2 Two-way ANOVA results for Firmness**

| Two-way ANOVA | Ordinary |  |  |  |  |
| --- | --- | --- | --- | --- | --- |
| Alpha | 0.05 |  |  |  |  |
|  |  |  |  |  |  |
| Source of Variation | % of total variation | P value | P value summary | Significant? |  |
| Interaction | 13.76 | 0.0008 | *** | Yes |  |
| Row Factor | 43.32 | <0.0001 | **** | Yes |  |
| Column Factor | 28.58 | <0.0001 | **** | Yes |  |
|  |  |  |  |  |  |
| ANOVA table | SS | DF | MS | F (DFn, DFd) | P value |
| Interaction | 13.10 | 20 | 0.6550 | F (20, 60) = 2.877 | P=0.0008 |
| Row Factor | 41.26 | 4 | 10.31 | F (4, 60) = 45.31 | P<0.0001 |
| Column Factor | 27.22 | 5 | 5.444 | F (5, 60) = 23.92 | P<0.0001 |
| Residual | 13.66 | 60 | 0.2276 |  |  |
|  |  |  |  |  |  |
| Data summary |  |  |  |  |  |
| Number of columns (Column Factor) | 6 |  |  |  |  |
| Number of rows (Row Factor) | 5 |  |  |  |  |
| Number of values | 90 |  |  |  |  |

**Table S3 Two-way ANOVA results for TSS**

| Two-way ANOVA | Ordinary |  |  |  |  |
| --- | --- | --- | --- | --- | --- |
| Alpha | 0.05 |  |  |  |  |
|  |  |  |  |  |  |
| Source of Variation | % of total variation | P value | P value summary | Significant? |  |
| Interaction | 6.839 | <0.0001 | **** | Yes |  |
| Row Factor | 27.09 | <0.0001 | **** | Yes |  |
| Column Factor | 62.50 | <0.0001 | **** | Yes |  |
|  |  |  |  |  |  |
| ANOVA table | SS | DF | MS | F (DFn, DFd) | P value |
| Interaction | 4.547 | 20 | 0.2273 | F (20, 60) = 5.751 | P<0.0001 |
| Row Factor | 18.01 | 4 | 4.502 | F (4, 60) = 113.9 | P<0.0001 |
| Column Factor | 41.56 | 5 | 8.311 | F (5, 60) = 210.2 | P<0.0001 |
| Residual | 2.372 | 60 | 0.03953 |  |  |
|  |  |  |  |  |  |
| Data summary |  |  |  |  |  |
| Number of columns (Column Factor) | 6 |  |  |  |  |
| Number of rows (Row Factor) | 5 |  |  |  |  |
| Number of values | 90 |  |  |  |  |

**Table S4 Two-way ANOVA results for TSS/TA**

| Two-way ANOVA | Ordinary |  |  |  |  |
| --- | --- | --- | --- | --- | --- |
| Alpha | 0.05 |  |  |  |  |
|  |  |  |  |  |  |
| Source of Variation | % of total variation | P value | P value summary | Significant? |  |
| Interaction | 0.6366 | <0.0001 | **** | Yes |  |
| Row Factor | 94.53 | <0.0001 | **** | Yes |  |
| Column Factor | 4.485 | <0.0001 | **** | Yes |  |
|  |  |  |  |  |  |
| ANOVA table | SS | DF | MS | F (DFn, DFd) | P value |
| Interaction | 157.3 | 20 | 7.867 | F (20, 60) = 5.463 | P<0.0001 |
| Row Factor | 23361 | 4 | 5840 | F (4, 60) = 4056 | P<0.0001 |
| Column Factor | 1108 | 5 | 221.7 | F (5, 60) = 153.9 | P<0.0001 |
| Residual | 86.39 | 60 | 1.440 |  |  |
|  |  |  |  |  |  |
| Data summary |  |  |  |  |  |
| Number of columns (Column Factor) | 6 |  |  |  |  |
| Number of rows (Row Factor) | 5 |  |  |  |  |
| Number of values | 90 |  |  |  |  |

**Table S5 Two-way ANOVA results for pH**

| Two-way ANOVA | Ordinary |  |  |  |  |
| --- | --- | --- | --- | --- | --- |
| Alpha | 0.05 |  |  |  |  |
|  |  |  |  |  |  |
| Source of Variation | % of total variation | P value | P value summary | Significant? |  |
| Interaction | 28.76 | <0.0001 | **** | Yes |  |
| Row Factor | 27.00 | <0.0001 | **** | Yes |  |
| Column Factor | 23.60 | <0.0001 | **** | Yes |  |
|  |  |  |  |  |  |
| ANOVA table | SS | DF | MS | F (DFn, DFd) | P value |
| Interaction | 0.2477 | 20 | 0.01238 | F (20, 60) = 4.182 | P<0.0001 |
| Row Factor | 0.2326 | 4 | 0.05814 | F (4, 60) = 19.63 | P<0.0001 |
| Column Factor | 0.2033 | 5 | 0.04066 | F (5, 60) = 13.73 | P<0.0001 |
| Residual | 0.1777 | 60 | 0.002961 |  |  |
|  |  |  |  |  |  |
| Data summary |  |  |  |  |  |
| Number of columns (Column Factor) | 6 |  |  |  |  |
| Number of rows (Row Factor) | 5 |  |  |  |  |
| Number of values | 90 |  |  |  |  |

**Table S6 Two-way ANOVA results for Vitamin C**

| Two-way ANOVA | Ordinary |  |  |  |  |
| --- | --- | --- | --- | --- | --- |
| Alpha | 0.05 |  |  |  |  |
|  |  |  |  |  |  |
| Source of Variation | % of total variation | P value | P value summary | Significant? |  |
| Interaction | 9.393 | <0.0001 | **** | Yes |  |
| Row Factor | 72.07 | <0.0001 | **** | Yes |  |
| Column Factor | 14.46 | <0.0001 | **** | Yes |  |
|  |  |  |  |  |  |
| ANOVA table | SS | DF | MS | F (DFn, DFd) | P value |
| Interaction | 251.9 | 20 | 12.60 | F (20, 60) = 6.913 | P<0.0001 |
| Row Factor | 1933 | 4 | 483.2 | F (4, 60) = 265.2 | P<0.0001 |
| Column Factor | 388.0 | 5 | 77.60 | F (5, 60) = 42.58 | P<0.0001 |
| Residual | 109.3 | 60 | 1.822 |  |  |
|  |  |  |  |  |  |
| Data summary |  |  |  |  |  |
| Number of columns (Column Factor) | 6 |  |  |  |  |
| Number of rows (Row Factor) | 5 |  |  |  |  |
| Number of values | 90 |  |  |  |  |

**Table S7 Two-way ANOVA results for TPC**

| Two-way ANOVA | Ordinary |  |  |  |  |
| --- | --- | --- | --- | --- | --- |
| Alpha | 0.05 |  |  |  |  |
|  |  |  |  |  |  |
| Source of Variation | % of total variation | P value | P value summary | Significant? |  |
| Interaction | 11.12 | <0.0001 | **** | Yes |  |
| Row Factor | 81.47 | <0.0001 | **** | Yes |  |
| Column Factor | 6.796 | <0.0001 | **** | Yes |  |
|  |  |  |  |  |  |
| ANOVA table | SS | DF | MS | F (DFn, DFd) | P value |
| Interaction | 1526 | 20 | 76.31 | F (20, 60) = 54.53 | P<0.0001 |
| Row Factor | 11178 | 4 | 2795 | F (4, 60) = 1997 | P<0.0001 |
| Column Factor | 932.5 | 5 | 186.5 | F (5, 60) = 133.3 | P<0.0001 |
| Residual | 83.96 | 60 | 1.399 |  |  |
|  |  |  |  |  |  |
| Data summary |  |  |  |  |  |
| Number of columns (Column Factor) | 6 |  |  |  |  |
| Number of rows (Row Factor) | 5 |  |  |  |  |
| Number of values | 90 |  |  |  |  |

**Table S8 Two-way ANOVA results for TFC**

| Two-way ANOVA | Ordinary |  |  |  |  |
| --- | --- | --- | --- | --- | --- |
| Alpha | 0.05 |  |  |  |  |
|  |  |  |  |  |  |
| Source of Variation | % of total variation | P value | P value summary | Significant? |  |
| Interaction | 13.51 | <0.0001 | **** | Yes |  |
| Row Factor | 81.87 | <0.0001 | **** | Yes |  |
| Column Factor | 4.324 | <0.0001 | **** | Yes |  |
|  |  |  |  |  |  |
| ANOVA table | SS | DF | MS | F (DFn, DFd) | P value |
| Interaction | 74.27 | 20 | 3.714 | F (20, 60) = 134.5 | P<0.0001 |
| Row Factor | 450.2 | 4 | 112.6 | F (4, 60) = 4076 | P<0.0001 |
| Column Factor | 23.78 | 5 | 4.755 | F (5, 60) = 172.2 | P<0.0001 |
| Residual | 1.657 | 60 | 0.02761 |  |  |
|  |  |  |  |  |  |
| Data summary |  |  |  |  |  |
| Number of columns (Column Factor) | 6 |  |  |  |  |
| Number of rows (Row Factor) | 5 |  |  |  |  |
| Number of values | 90 |  |  |  |  |
|  |  |  |  |  |  |

**Table S9 Two-way ANOVA results for L***

| Two-way ANOVA | Ordinary |  |  |  |  |
| --- | --- | --- | --- | --- | --- |
| Alpha | 0.05 |  |  |  |  |
|  |  |  |  |  |  |
| Source of Variation | % of total variation | P value | P value summary | Significant? |  |
| Interaction | 26.21 | 0.0301 | * | Yes |  |
| Row Factor | 21.49 | <0.0001 | **** | Yes |  |
| Column Factor | 10.74 | 0.0148 | * | Yes |  |
|  |  |  |  |  |  |
| ANOVA table | SS | DF | MS | F (DFn, DFd) | P value |
| Interaction | 631.3 | 20 | 31.57 | F (20, 60) = 1.892 | P=0.0301 |
| Row Factor | 517.6 | 4 | 129.4 | F (4, 60) = 7.757 | P<0.0001 |
| Column Factor | 258.7 | 5 | 51.74 | F (5, 60) = 3.102 | P=0.0148 |
| Residual | 1001 | 60 | 16.68 |  |  |
|  |  |  |  |  |  |
| Data summary |  |  |  |  |  |
| Number of columns (Column Factor) | 6 |  |  |  |  |
| Number of rows (Row Factor) | 5 |  |  |  |  |
| Number of values | 90 |  |  |  |  |
|  |  |  |  |  |  |

**Table S10 Two-way ANOVA results for a***

| Two-way ANOVA | Ordinary |  |  |  |  |
| --- | --- | --- | --- | --- | --- |
| Alpha | 0.05 |  |  |  |  |
|  |  |  |  |  |  |
| Source of Variation | % of total variation | P value | P value summary | Significant? |  |
| Interaction | 15.53 | 0.0314 | * | Yes |  |
| Row Factor | 16.00 | <0.0001 | **** | Yes |  |
| Column Factor | 43.69 | <0.0001 | **** | Yes |  |
|  |  |  |  |  |  |
| ANOVA table | SS | DF | MS | F (DFn, DFd) | P value |
| Interaction | 3.134 | 20 | 0.1567 | F (20, 60) = 1.880 | P=0.0314 |
| Row Factor | 3.227 | 4 | 0.8069 | F (4, 60) = 9.683 | P<0.0001 |
| Column Factor | 8.814 | 5 | 1.763 | F (5, 60) = 21.15 | P<0.0001 |
| Residual | 5.000 | 60 | 0.08333 |  |  |
|  |  |  |  |  |  |
| Data summary |  |  |  |  |  |
| Number of columns (Column Factor) | 6 |  |  |  |  |
| Number of rows (Row Factor) | 5 |  |  |  |  |
| Number of values | 90 |  |  |  |  |

**Table S11 Two-way ANOVA results for b***

| Two-way ANOVA | Ordinary |  |  |  |  |
| --- | --- | --- | --- | --- | --- |
| Alpha | 0.05 |  |  |  |  |
|  |  |  |  |  |  |
| Source of Variation | % of total variation | P value | P value summary | Significant? |  |
| Interaction | 33.75 | 0.0133 | * | Yes |  |
| Row Factor | 12.51 | 0.0067 | ** | Yes |  |
| Column Factor | 5.969 | 0.2036 | ns | No |  |
|  |  |  |  |  |  |
| ANOVA table | SS | DF | MS | F (DFn, DFd) | P value |
| Interaction | 342.1 | 20 | 17.10 | F (20, 60) = 2.119 | P=0.0133 |
| Row Factor | 126.8 | 4 | 31.70 | F (4, 60) = 3.927 | P=0.0067 |
| Column Factor | 60.50 | 5 | 12.10 | F (5, 60) = 1.499 | P=0.2036 |
| Residual | 484.2 | 60 | 8.071 |  |  |
|  |  |  |  |  |  |
| Data summary |  |  |  |  |  |
| Number of columns (Column Factor) | 6 |  |  |  |  |
| Number of rows (Row Factor) | 5 |  |  |  |  |
| Number of values | 90 |  |  |  |  |
|  |  |  |  |  |  |

**Table S12 Two-way ANOVA results for Hue angle**

| Two-way ANOVA | Ordinary |  |  |  |  |
| --- | --- | --- | --- | --- | --- |
| Alpha | 0.05 |  |  |  |  |
|  |  |  |  |  |  |
| Source of Variation | % of total variation | P value | P value summary | Significant? |  |
| Interaction | 38.24 | 0.0005 | *** | Yes |  |
| Row Factor | 7.268 | 0.0313 | * | Yes |  |
| Column Factor | 16.26 | 0.0006 | *** | Yes |  |
|  |  |  |  |  |  |
| ANOVA table | SS | DF | MS | F (DFn, DFd) | P value |
| Interaction | 0.05878 | 20 | 0.002939 | F (20, 60) = 3.000 | P=0.0005 |
| Row Factor | 0.01117 | 4 | 0.002793 | F (4, 60) = 2.851 | P=0.0313 |
| Column Factor | 0.02499 | 5 | 0.004998 | F (5, 60) = 5.102 | P=0.0006 |
| Residual | 0.05877 | 60 | 0.0009795 |  |  |
|  |  |  |  |  |  |
| Data summary |  |  |  |  |  |
| Number of columns (Column Factor) | 6 |  |  |  |  |
| Number of rows (Row Factor) | 5 |  |  |  |  |
| Number of values | 90 |  |  |  |  |

**Table S13 Two-way ANOVA results for ∆E**

| Two-way ANOVA | Ordinary |  |  |  |  |
| --- | --- | --- | --- | --- | --- |
| Alpha | 0.05 |  |  |  |  |
|  |  |  |  |  |  |
| Source of Variation | % of total variation | P value | P value summary | Significant? |  |
| Interaction | 23.06 | 0.1294 | ns | No |  |
| Row Factor | 21.19 | 0.0002 | *** | Yes |  |
| Column Factor | 8.489 | 0.0710 | ns | No |  |
|  |  |  |  |  |  |
| ANOVA table | SS | DF | MS | F (DFn, DFd) | P value |
| Interaction | 613.1 | 20 | 30.65 | F (20, 60) = 1.464 | P=0.1294 |
| Row Factor | 563.3 | 4 | 140.8 | F (4, 60) = 6.727 | P=0.0002 |
| Column Factor | 225.6 | 5 | 45.13 | F (5, 60) = 2.156 | P=0.0710 |
| Residual | 1256 | 60 | 20.93 |  |  |
|  |  |  |  |  |  |
| Data summary |  |  |  |  |  |
| Number of columns (Column Factor) | 6 |  |  |  |  |
| Number of rows (Row Factor) | 5 |  |  |  |  |
| Number of values | 90 |  |  |  |  |

**Table S14 Two-way ANOVA results for TVC**

| Two-way ANOVA | Ordinary |  |  |  |  |
| --- | --- | --- | --- | --- | --- |
| Alpha | 0.05 |  |  |  |  |
|  |  |  |  |  |  |
| Source of Variation | % of total variation | P value | P value summary | Significant? |  |
| Interaction | 63.07 | <0.0001 | **** | Yes |  |
| Row Factor | 18.83 | <0.0001 | **** | Yes |  |
| Column Factor | 18.07 | <0.0001 | **** | Yes |  |
|  |  |  |  |  |  |
| ANOVA table | SS | DF | MS | F (DFn, DFd) | P value |
| Interaction | 49836100337536 | 20 | 2491805016877 | F (20, 60) = 7497 | P<0.0001 |
| Row Factor | 14880995820718 | 4 | 3720248955179 | F (4, 60) = 11194 | P<0.0001 |
| Column Factor | 14282457845409 | 5 | 2856491569082 | F (5, 60) = 8595 | P<0.0001 |
| Residual | 19941268533 | 60 | 332354476 |  |  |
|  |  |  |  |  |  |
| Data summary |  |  |  |  |  |
| Number of columns (Column Factor) | 6 |  |  |  |  |
| Number of rows (Row Factor) | 5 |  |  |  |  |
| Number of values | 90 |  |  |  |  |
|  |  |  |  |  |  |
|  |  |  |  |  |  |
|  |  |  |  |  |  |
|  |  |  |  |  |  |

**Table S15 Two-way ANOVA results for TMYC**

| Two-way ANOVA | Ordinary |  |  |  |  |
| --- | --- | --- | --- | --- | --- |
| Alpha | 0.05 |  |  |  |  |
|  |  |  |  |  |  |
| Source of Variation | % of total variation | P value | P value summary | Significant? |  |
| Interaction | 29.52 | <0.0001 | **** | Yes |  |
| Row Factor | 56.77 | <0.0001 | **** | Yes |  |
| Column Factor | 13.54 | <0.0001 | **** | Yes |  |
|  |  |  |  |  |  |
| ANOVA table | SS | DF | MS | F (DFn, DFd) | P value |
| Interaction | 9453 | 20 | 472.6 | F (20, 60) = 525.1 | P<0.0001 |
| Row Factor | 18182 | 4 | 4545 | F (4, 60) = 5051 | P<0.0001 |
| Column Factor | 4337 | 5 | 867.5 | F (5, 60) = 963.9 | P<0.0001 |
| Residual | 54.00 | 60 | 0.9000 |  |  |
|  |  |  |  |  |  |
| Data summary |  |  |  |  |  |
| Number of columns (Column Factor) | 6 |  |  |  |  |
| Number of rows (Row Factor) | 5 |  |  |  |  |
| Number of values | 90 |  |  |  |  |
|  |  |  |  |  |  |
|  |  |  |  |  |  |
